# SARS-CoV-2 infection of airway cells causes intense viral and cell shedding, two spreading mechanisms affected by IL-13

**DOI:** 10.1073/pnas.2119680119

**Published:** 2022-03-30

**Authors:** Cameron B. Morrison, Caitlin E. Edwards, Kendall M. Shaffer, Kenza C. Araba, Jason A. Wykoff, Danielle R. Williams, Takanori Asakura, Hong Dang, Lisa C. Morton, Rodney C. Gilmore, Wanda K. O’Neal, Richard C. Boucher, Ralph S. Baric, Camille Ehre

**Affiliations:** ^a^Marsico Lung Institute, The University of North Carolina at Chapel Hill, Chapel Hill, NC 27599;; ^b^Department of Epidemiology, The University of North Carolina at Chapel Hill, Chapel Hill, NC 27599;; ^c^Department of Microbiology and Immunology, The University of North Carolina at Chapel Hill, Chapel Hill, NC 27599;; ^d^Department of Pediatrics/Pediatric Pulmonology, The University of North Carolina at Chapel Hill, Chapel Hill, NC 27599

**Keywords:** SARS-CoV-2, human airway epithelial cells, infection, asthma, IL-13 cytokine

## Abstract

Muco-obstructive lung diseases are typically associated with high risks of COVID-19 severity; however, allergic asthma showed reduced susceptibility. To investigate viral spread, primary human airway epithelial (HAE) cell cultures were infected with severe acute respiratory syndrome coronavirus 2 (SARS-CoV-2), and host–virus interactions were examined via electron microscopy, immunohistochemistry, RNA in situ hybridization, and gene expression analyses. In HAE cell cultures, angiotensin-converting enzyme 2 (ACE2) expression governed cell tropism and viral load and was up-regulated by infection. Electron microscopy identified intense viral egress from infected ciliated cells and severe cytopathogenesis, culminating in the shedding of ciliated cells packed with virions, providing a large viral reservoir for spread and transmission. Intracellular stores of MUC5AC, a major airway mucin involved in asthma, were rapidly depleted, likely to trap viruses. To mimic asthmatic airways, HAE cells were treated with interleukin-13 (IL-13), which reduced viral titers, viral messenger RNA, and cell shedding, and significantly diminished the number of infected cells. Although mucus hyperproduction played a shielding role, IL-13–treated cells maintained a degree of protection despite the removal of mucus. Using Gene Expression Omnibus databases, bulk RNA-sequencing analyses revealed that IL-13 up-regulated genes controlling glycoprotein synthesis, ion transport, and antiviral processes (albeit not the typical interferon-induced genes) and down-regulated genes involved in cilial function and ribosomal processing. More precisely, we showed that IL-13 reduced ACE2 expression, intracellular viral load, and cell-to-cell transmission while increasing the cilial keratan sulfate coating. In conclusion, intense viral and cell shedding caused by SARS-CoV-2 infection was attenuated by IL-13, which affected viral entry, replication, and spread.

## Significance

Gaining insights into severe acute respiratory syndrome coronavirus 2 (SARS-CoV-2) high transmissibility and the role played by inflammatory mediators in viral proliferation are critical to investigating new therapeutic targets against COVID-19. Electron microscopy reveals important SARS-CoV-2 features, including the combination of large, rapidly released viral clusters and the massive shedding of epithelial cells packed with virions. Interleukin-13 (IL-13), a Th2 cytokine up-regulated in allergic asthma and associated with less severe COVID-19, protects against SARS-CoV-2 viral and cell shedding. Using gene expression analyses and biochemical assays, IL-13 is shown to affect viral entry, replication, and cell-to-cell transmission. Given the broad spectrum of COVID-19 clinical symptoms, it is important to elucidate intrinsic factors that modulate viral load and spreading mechanisms.

Severe acute respiratory syndrome coronavirus 2 (SARS-CoV-2), the virus causing COVID-19, is the third coronavirus outbreak affecting the human population since the beginning of the 21st century and was preceded by SARS-CoV in 2002 and Middle East respiratory syndrome (MERS)–CoV in 2012 (1–3). More than 2 y after the initial outbreak, SARS-CoV-2 continues to be actively transmitted in the human population and has already killed >5 million people. The clinical symptoms of COVID-19 vary greatly between infected individuals, ranging from asymptomatic disease to severe respiratory illness and even death in nearly 2% of cases (4, 5). Despite similarities in transmission, infection, and clinical symptoms with other human coronaviruses, the elevated transmissibility and global burden posed by SARS-CoV-2 warrant further investigation into its tropism, pathogenesis, and spreading mechanism.

SARS-CoV-2 utilizes the angiotensin-converting enzyme 2 (ACE2) receptor and a transmembrane serine protease (TMPRSS2) to enter host cells, and both proteins are coexpressed in respiratory epithelia (6, 7). ACE2 expression has been detected in ciliated and goblet cells, two important cell types involved in airway clearance (8, 9). Using human airway epithelial (HAE) cell models, we have shown that SARS-CoV-2 efficiently infected cells from the upper airways, suggesting that the nose and large airways are preferred sites for viral transmission and replication (10, 11). Furthermore, scanning electron microscopy (SEM) confirmed direct interactions between SARS-CoV-2 viruses and ciliated epithelial cells (12).

In the lungs, ciliated cells coordinate the movement of secretions to clear inhaled particles, a process referred to as mucociliary transport (MCT). Thus, viral infections targeting this particular cell type can affect airway clearance. Tropism for ciliated cells has been reported for other respiratory viruses (e.g., respiratory syncytial virus, influenza, and other coronaviruses), which was associated with cell shedding (13–15). Another important player in viral infection is the glycocalyx coating the periciliary (PCL) region to provide a barrier while facilitating cilia beating (16, 17). In response to infection, goblet cells increase the secretion of mucins, the large polymeric glycoproteins responsible for the viscoelastic properties of mucus (18, 19). MUC5AC, a major gel-forming mucin expressed in the lungs, is secreted in small amounts in healthy individuals and is up-regulated during respiratory infections, suggesting a protective role against pathogens (20–23). In diseases such as chronic obstructive pulmonary disease (COPD) and asthma, MUC5AC is up-regulated, and has been associated with reduced MCT (24–26). COPD patients infected with SARS-CoV-2 are at higher risk of severe clinical outcomes, but, for asthmatic patients, the data are more contradictory (27–31). The variability within the asthmatic population may originate from pathophysiologic differences based on Th2-low (nonallergic) and Th2-high (allergic) inflammatory profiles, with allergic asthma being protective (32, 33). IL-13, a type 2 cytokine associated with allergic asthma, has been shown to increase MUC5AC secretion (34, 35) and down-regulate ACE2 expression (30, 36), and the effects of IL-13 on SARS-CoV-2 infection are only beginning to be explored (37).

In this study, we infected HAE cell cultures with SARS-CoV-2 and examined the fate of infected airway cells using fluorescent labeling, combined with SEM and transmission electron microscopy (TEM). These approaches characterized the fate of infected cells by examining the mode of virion cellular release and the detachment of infected cells. In addition, we investigated whether IL-13 protects against viral spread. The effects of IL-13 on airway cultures were determined by measuring viral titers, viral gene replication, and epithelial cell damage. Mucus hyperproduction provided a physical barrier; however, after removing mucus either by cell washing or using MUC5AC knockout (KO) cells, lower viral loads were maintained in IL-13–treated cells. To investigate how IL-13 modified host antiviral responses, the effects of IL-13 administration on gene expression in HAE cultures were analyzed from three independent bulk RNA-sequencing studies available on the Gene Expression Omnibus (GEO) database. These analyses revealed that critical processes for viral entry, replication, and spread, pertinent to asthma and COVID-19 severity, were affected by IL-13 treatment.

## Results

### Cell Tropism and Epithelial Response.

**Fig. 1.**
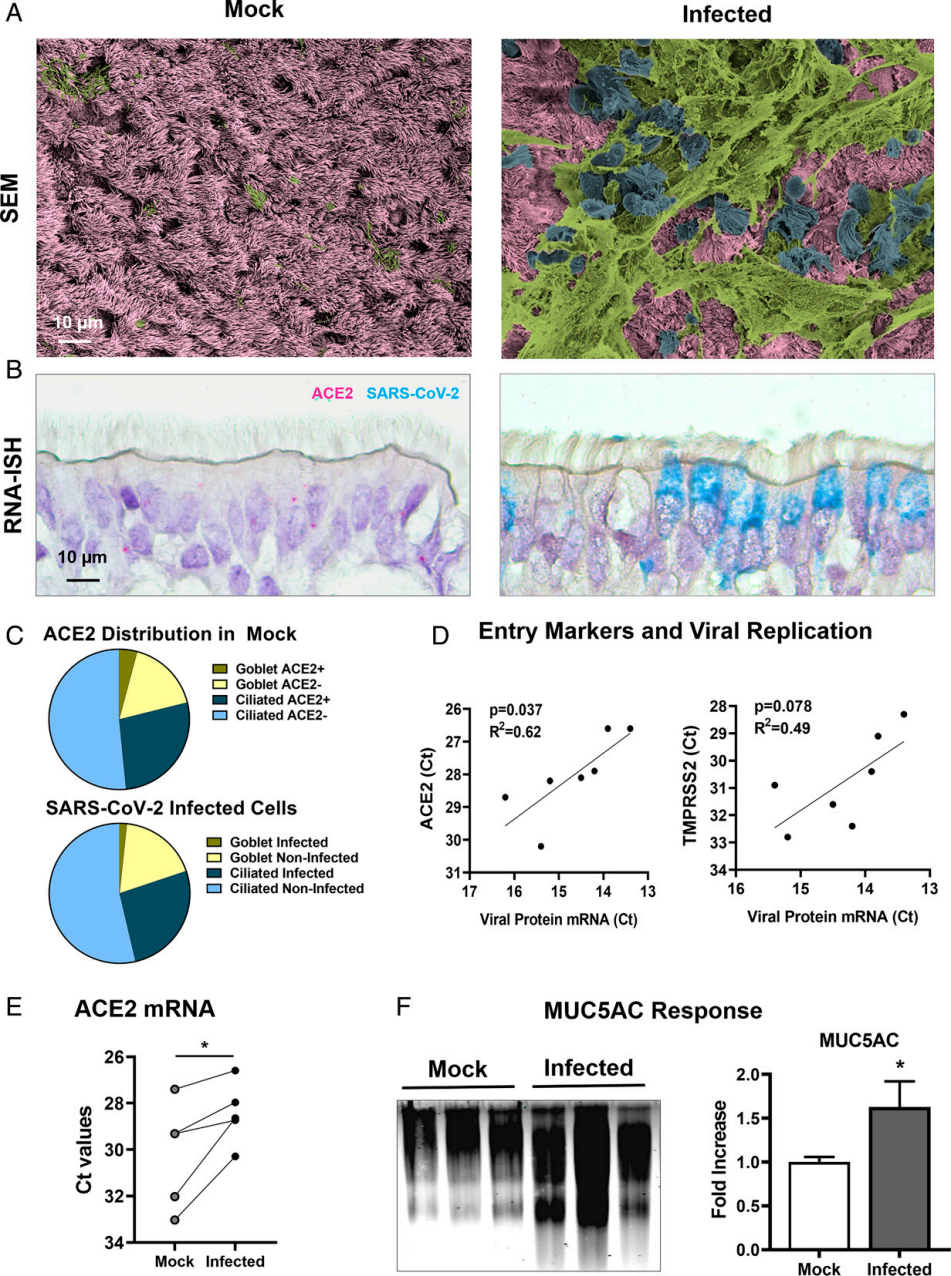
Viral entry markers and mucin secretion in mock and SARS-CoV-2–infected HAE cell cultures. Mock and infected (MOI = 0.5) HAE cell cultures were fixed or washed and processed for mRNA extraction. (*A*) En face images of mock and infected HAE cultures at 96 hpi via scanning electron microscope. Images were colorized using Adobe Photoshop to identify healthy cells (pink), mucus (green), and cells in the process of anoikis (blue). (*B*) RNA-ISH on mock and infected HAE cells at 48 hpi using an ACE2 mRNA probe (magenta) and a SARS-CoV-2 mRNA probe (cyan). (*C*) Pie charts showing the percentage of ciliated and goblet cells expressing the ACE2 receptor (*Top*) and the distribution of SARS-CoV-2–infected cells at 48 hpi (*Bottom*) via RNA-ISH. Cells exhibiting at least one dot per cell were counted as positive; *n* = 11. (*D*) Correlation between viral entry markers ACE2 and TMPRSS2 mRNA at 0 hpi and nucleocapsid protein mRNA in infected cultures at 72 hpi, as indicated by cross-point threshold (Ct); *n* = 3 to 6 inserts per donor. (*E*) Change in ACE2 mRNA expression in response to infection, comparing mock and infected HAE cells at 72 hpi; *n* = 3 to 6 inserts per donors. (*F*) Increased MUC5AC secretion in response to SARS-CoV-2 infection. Cell washings from mock and infected cultures were collected at 72 hpi and analyzed by mucin Western blot, as shown in a representative image. Graph shows the relative change in MUC5AC signal intensity in mock vs. infected HAE cultures; *n* = 8. *P < 0.05.

Mock and infected HAE cultures were examined via SEM to characterize the topography of the airway epithelium at 96 h postinfection (hpi) (Fig. 1*A* and *SI Appendix*, Fig. S1). Mock cultures displayed a homogenous appearance, which consisted of a layer of cilia with interspersed goblet cells lining the apical surfaces. In contrast, infected cultures exhibited substantial epithelial damage with mucus sheets covering cells in the process of anoikis. In healthy airways, apical cells exposed to luminal pathogens mostly consist of ciliated (∼80%) and secretory (∼20%) cells (38). RNA in situ hybridization (RNA-ISH) of mock cultures revealed that 31% of apical cells expressed the SARS-CoV-2 receptor and that ACE2-positive cells were divided between ciliated and goblet cells at roughly a 6:1 ratio (Fig. 1 *B* and *C* and *SI Appendix*, Fig. S2). RNA-ISH using the SARS-CoV-2 probe revealed a strong tropism for ciliated cells, with a 16:1 ratio of infected ciliated to goblet cells at 48 hpi.

To further investigate the role of viral entry markers, qPCR was performed on seven individual lung donors and showed a positive correlation between ACE2 messenger RNA (mRNA) expression (and TMPRSS2 to a lesser degree) and the intracellular viral genomic RNA (or viral load) at 72 hpi (Fig. 1*D*). These results indicate that ACE2 receptor density plays a key role in the ability of the virus to spread. Importantly, ACE2 expression was increased by infection, a process further intensifying viral proliferation (Fig. 1*E*). RNA-ISH revealed a slight and proportional increase in the number of ciliated and goblet cells expressing ACE2 following infection, which was associated with more copies of mRNA per cell, especially in ciliated cells (*SI Appendix*, Fig. S2). In response to infection, MUC5AC secretion was increased by 1.63-fold (Fig. 1*F*), a common airway epithelial response to trap pathogens.

### Intense Viral and Cell Shedding.

**Fig. 2.**
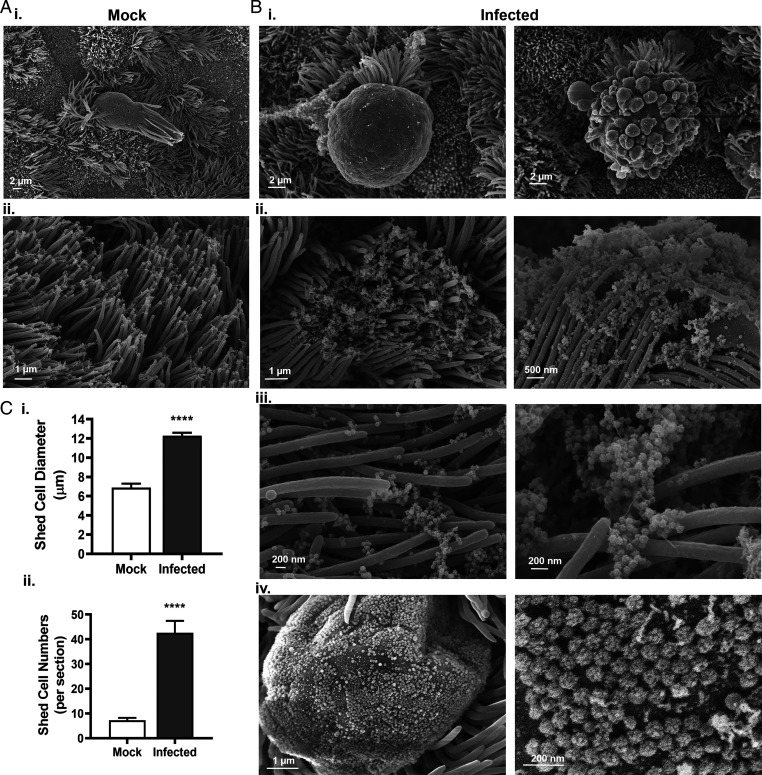
Intense viral and cell shedding from SARS-CoV-2–infected HAE cells. HAE cells were incubated with PBS (mock) or infected with SARS-CoV-2 (MOI = 0.5) and processed for SEM. (*A*) Representative images of a mock culture exhibiting sporadic ciliated shedding (*i*) and loose cilia tips (*ii*). (*B*) Representative images of infected cultures at 96 hpi showing ballooning shed cells (*i*), cilia tips encased with virions (*ii*), clusters of virions emerging between cilia shafts (*iii*), and cell protrusion with virions scattered across the cell membrane (*iv*). (*C*) Quantitative analyses of shed cell width (*i*) and number of shed cells per insert at 24 hpi (*ii*); *n* = 18 infected HAE cultures from six donors. ****P < 0.0001.

As shown in Fig. 2, SEM images revealed that epithelial damage in infected HAE cell cultures typically consisted of swollen, shed ciliated cells surrounded by threads of mucus. In mock cultures, ciliated cell anoikis was observed at a low incidence (<10 cells per insert), while the number of shed cells in infected cultures was fourfold higher within 24 hpi (i.e., prior to the onset of symptoms in infected individuals) (Fig. 2*C*). In mock cultures, detached ciliated cells displayed a columnar appearance with a standard 7-μm width, while shed cells in infected cultures exhibited irregular shapes and sizes, with aberrant membrane protuberances and a width ranging from 10 μm to 15 μm. High-resolution images revealed intense viral egress from shed ciliated cells (*SI Appendix*, Figs. S3–S5). In addition, massive viral shedding was observed from plasma membrane protrusions and from epithelial cells crowned with disorganized cilia, releasing large clusters of virions (>200 virus particles in a 2-µm radius) between the microvilli and the cilial shafts from a single cell. Detailed examination of these ∼100-nm particles confirmed the presence of viral spikes characteristic of coronaviruses. Hence, a hallmark of SARS-CoV-2–infected cultures was intense viral egress from ciliated cells accompanied by anoikis.

### Cytopathology Caused by SARS-CoV-2 Infection.

**Fig. 3.**
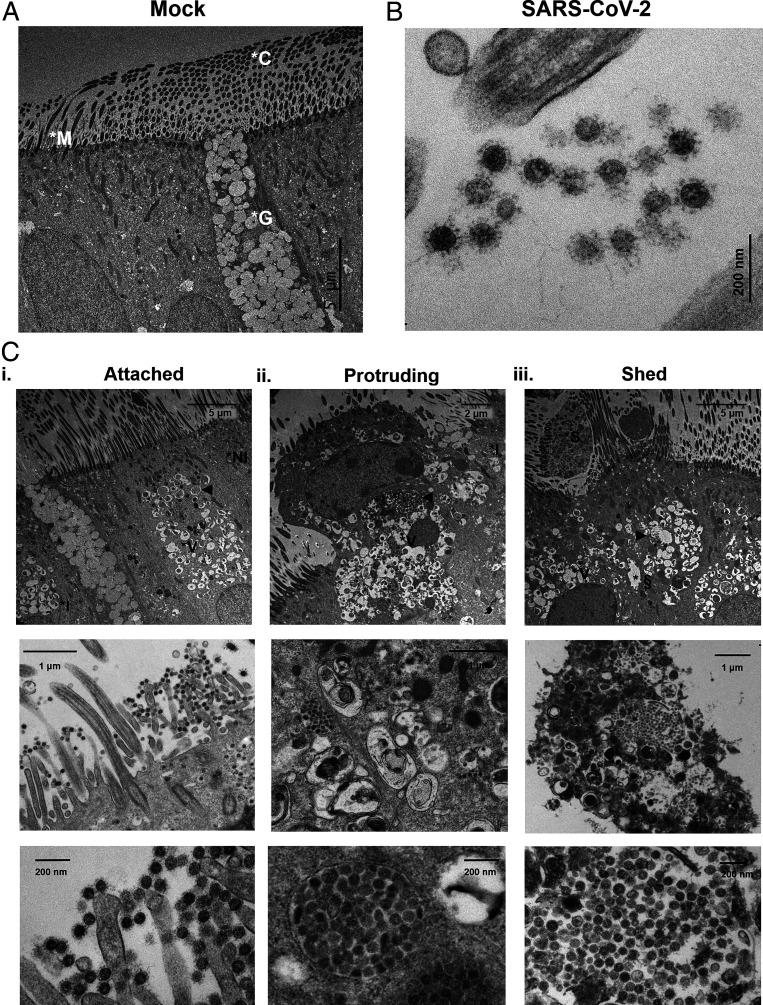
Three cytopathologic stages of SARS-CoV-2–infected ciliated cells. (*A*) TEM image of a mock HAE cell culture depicting a goblet cell (packed with mucin granules) amid ciliated cells. (*B*) High-magnification TEM images of an infected HAE culture at 96 hpi showing a cluster of SARS-CoV-2 viruses between cilial shafts. (*C*) TEM images reveal three stages for SARS-CoV-2–infected ciliated cells: attached (*i*), protruding (*ii*), and shed stages (*iii*). (*Top*) Low-, (*Middle*) middle-, and (*Bottom*) high-magnification images of infected cells at the different stages. In the attached stage, the cytoplasm of infected cells was occupied by vacuoles, and the nucleus remained intact. In the protruding stage, infected cells expanded outwardly, vacuoles were packed with virions, and nuclei show signs of pyknosis. In the shed stage, ciliated cells were fully detached from the epithelium, the intracellular vacuoles were large and tightly packed with virions, the nuclei showed signs of karyorrhexis, and the cell membrane was disrupted, facilitating the release of contents into the lumen. High-magnification images confirm the presence of viral spikes on high-contrast, ∼100-nm spherical structures. *G, goblet cell; *C, cilium; *M, microvilli; *I, infected cell; *NI, noninfected cell; *V, vacuole; ▸, SARS-CoV-2 virions; *S, shed cell; and *§, ruptured cellular junctions; *n* = 2 inserts from four donors.

TEM provided cross-sectional, high-magnification views of airway cells, which facilitated the detection of intracellular virions and confirmed that the majority of SARS-CoV-2–infected cells were ciliated cells (Fig. 3). A small fraction (3 out of 58 infected cells or ∼5%) of infected cells were goblet cells (*SI Appendix*, Fig. S6). This imaging approach validated our SEM observations and confirmed that the presence of clustered virions occupying the extracellular space (e.g., surrounding microvilli, cilia, and membrane protrusions) was attributable to intense viral replication and egress from infected cells. In addition, TEM showed the extent of intracellular damage caused by SARS-CoV-2 replication and revealed three stages of the SARS-CoV-2–infected cell fate, accompanied by progressive nuclear destruction or karyolysis. The attached stage was characterized by ciliated cells that remained within the epithelium and were releasing viruses from intracellular virion-containing vacuoles of various sizes and densities (Fig. 3 *C*, *i* and *SI Appendix*, Fig. S7). Note that the formation of vacuoles was specific to ciliated cells, perhaps owing to the fact that goblet cells possess the entire machinery for granule release. The protruding stage was characterized by the initiation of cell detachment as infected cells swelled and expanded outwardly, and cellular junctions began to rupture (Fig. 3 *C*, *ii*). The shed stage was characterized by a complete detachment of rounded cells that enclosed large, virion-filled vacuoles, a process that was associated with advanced cellular degradation, including features of apoptosis and secondary necrosis (e.g., denser cytoplasm, packed organelles, karyorrhectic nuclei, rupture of cell membranes, and release of intracellular contents) (Fig. 3 *C*, *iii*). As cells detached from the epithelium, the size of virion-filled vacuoles increased, which was coupled with progressive disintegration of the plasma membrane, providing a robust spreading/transmission mechanism for SARS-CoV-2.

### IL-13 Treatment Reduced Viral Replication and Cell Shedding.

**Fig. 4.**
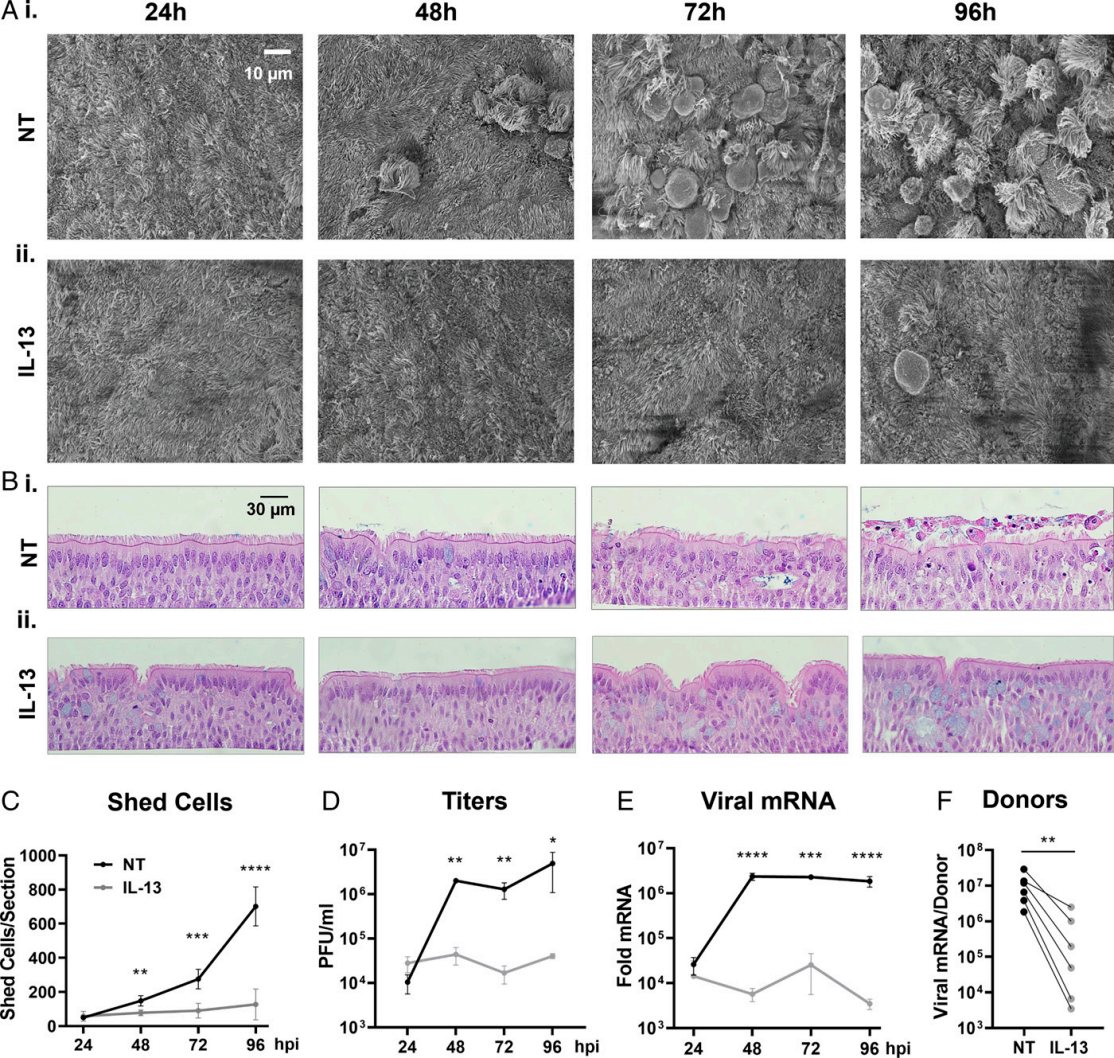
Effects of IL-13 treatment on viral replication and cell shedding. Three days prior to infection, HAE cultures were divided into two groups, the NT/PBS group (NT) and the IL-13–treated group (IL-13) to which 1 ng/mL of IL-13 was added via the basolateral side. On day 0, cells were infected with SARS-CoV-2 (MOI = 0.5) and processed at 24, 48, 72, and 96 hpi for histology, viral titers, and mRNA extraction. (*A*) Representative SEM en face views of infected HAE cells at 24, 48, 72, and 96 hpi for NT (*i*) and IL-13–treated (*ii*) cells. Viral shedding, cell swelling, and detachment was observed at 24 hpi to 48 hpi in the NT group, while limited epithelial damage was noticed up to 96 hpi in the IL-13 group. (*B*) Representative H&E images of infected HAE cells at 24, 48, 72, and 96 hpi for NT (*i*) and IL-13–treated (*ii*) cells. (*C*) Graph showing the number of shed cells per histological section for NT (black) and IL-13 (gray) groups; *n* = 4 sections per group. (*D*) Progression of viral titers in PFU per milliliter over time in NT and IL-13–treated cells; *n* = 3 inserts per time point per group. (*E*) Relative change in the nucleocapsid gene mRNA expression for both groups. Fold change shown as 2^^-ΔΔCt^; *n* = 3 cultures per time point per group. (*F*) Paired graph for the change in nucleocapsid mRNA expression at 96 hpi in response to IL-13 treatment for six donors; *n* = 3 to 6 inserts per group per donor. *P < 0.05, **P < 0.01, ***P < 0.001, ****P < 0.0001.

Th2-high asthmatics producing high levels of IL-13 in their lungs appear to have reduced susceptibility to severe COVID-19 (28, 29, 39). To examine the effects of IL-13 on SARS-CoV-2 infection, HAE cultures were pretreated with IL-13 for 3 d prior to infection, and treatment was maintained throughout the infection period. Cells from both nontreated (NT) and IL-13–treated (IL-13) HAE cultures were inoculated with SARS-CoV-2 and monitored over a 4-d period for viral replication and anoikis. SEM revealed that IL-13 treatment prevented epithelial damage and cell shedding when compared to cultures under NT conditions for 96 hpi (Fig. 4*A* and *SI Appendix*, Fig. S8). Histological sections acquired at 24, 48, 72, and 96 hpi and stained with hematoxylin and eosin (H&E) revealed striking differences between the two groups, including goblet cell hyperplasia in IL-13–treated cultures (Fig. 4*B*). In the NT group, epithelial cell damage increased over time, starting with uneven cell sizes/shapes at 48 hpi and progressing to substantial cell shedding (∼700 shed cells per insert) at 96 hpi. In contrast, IL-13–treated ciliated cells were well preserved over the course of the study, and the number of shed cells was approximately sixfold lower in the treatment group compared to the NT group at 96 hpi (Fig. 4*C*). The improved culture appearance in the IL-13 group correlated with a significant drop in viral titers (10^6^ vs. 10^4^ PFU/mL for NT and IL-13, respectively) and a two-log reduction in nucleocapsid protein mRNA expression (Fig. 4 *D* and *E*). The effects of IL-13 treatment on SARS-CoV-2 replication were tested on six additional donors and showed a significant decrease in nucleocapsid mRNA for all treated versus NT cells (Fig. 4*F*). Interestingly, IL-13 provided varying levels of protection for each individual donor, suggesting that patient genetic background and/or cytokine profile may play a role in COVID-19 disease heterogeneity (10).

### IL-13 Increased MUC5AC Production and Decreased Infected Cell Number.

**Fig. 5.**
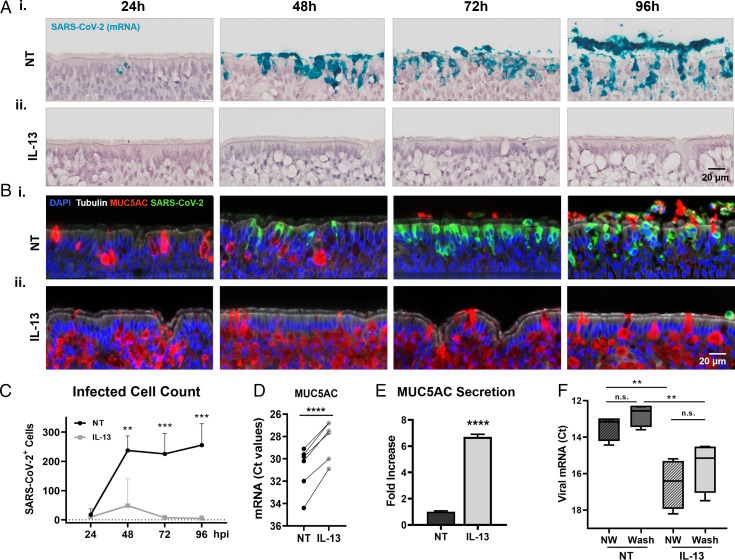
Effects of IL-13 treatment on infection and mucus production. (*A*) Representative RNA-ISH images of infected HAE cell cultures at 24, 48, 72, and 96 hpi labeled with a SARS-CoV-2 mRNA probe (cyan) for NT (*i*) and IL-13–treated (*ii*) cells. (*B*) Representative images of infected HAE cell cultures at 24, 48, 72, and 96 hpi immunostained with α-tubulin (white), SARS-CoV-2 (green), MUC5AC (red), and DAPI (blue) showing NT (*i*) and IL-13–treated (*ii*) cells. (*C*) Number of SARS-CoV-2–positive cells per histological section in infected HAE cell cultures for NT (black) and IL-13–treated (gray) cells. Note that shed cells were not included in the count; *n* = 4 sections per time point per group. (*D*). Relative changes in MUC5AC mRNA expression in response to IL-13 treatment in mock HAE cells from seven donors. Values for mRNA were normalized to glyceraldehyde-3-phosphate dehydrogenase; *n* = 3 to 6 inserts per group per donor. (*E*) Graph indicates normalized MUC5AC signal intensity from NT and IL-13–treated cell washings as measured by mucin Western blotting; *n* = 3 per group. (*F*) Graph showing the effect of apical mucus removal on viral replication in NT and IL-13–treated cultures. Apical surfaces were not washed (NW) or washed 3 times for 1 h with PBS (Wash) to remove mucus prior to infection. Nucleocapsid mRNA Ct values were measured at 72 hpi. **P < 0.01, ***P < 0.001, ****P < 0.0001, not significant (n.s.).

To visualize the progression of SARS-CoV-2 infection in NT and IL-13–treated cultures, RNA-ISH, immunohistochemistry (IHC), alcian blue periodic acid-Schiff (AB-PAS), and terminal deoxynucleotidyltransferase-mediated dUTP nick end labeling (TUNEL) staining were used to identify cilia, mucus, virions, and apoptotic cells at 24, 48, 72, and 96 hpi (Fig. 5 *A* and *B* and *SI Appendix*, Figs. S9 and S10). Goblet cells, detected by AB-PAS staining and MUC5AC IHC, were inserted between and underneath a layer of ciliated cells. At the start of infection (i.e., 3 d from the start of IL-13 treatment), goblet cells had increased in size and number in response to the cytokine (Figs. 4 *A* and *B* and 5 *A* and *B* and *SI Appendix*, Figs. S9 and S10). Goblet cell hyperplasia was accompanied by an increase in the overall height of the pseudostratified epithelium. Note that the apical cell layer that was initially exposed to viruses remained heavily populated by ciliated cells despite treatment. In fact, the total number of ciliated cells at the cell surface was unchanged by IL-13, and newly differentiated goblet cells reached the cell surface via a duct-like structure to release mucin granules (*SI Appendix*, Fig. S9). Following infection in the NT group, AB-PAS and MUC5AC intracellular signals decreased and redistributed entirely to the extracellular compartment within 3 d, suggesting mucin secretion as an adaptive response of the epithelium to infection. In parallel, SARS-CoV-2 intracellular mRNA and protein signals increased over time in the NT group, and the majority of shed cells exhibited SARS-CoV-2 signals in the later time points. TUNEL staining showed that apoptotic cell numbers increased over time in the NT group for both attached and shed cells (*SI Appendix*, Fig. S10). In contrast, in the IL-13 group, intracellular AB-PAS and MUC5AC intracellular signals remained high, while SARS-CoV-2 and TUNEL staining remained low for all time points. The number of SARS-CoV-2–positive cells (excluding shed cells) was quantified in both groups and showed a dramatic reduction of SARS-CoV-2–positive cells in IL-13 (<50 cells per section) compared to NT cultures (>250 cells per section) at 96 hpi (Fig. 5*C*); qPCR was used to examine the effects of IL-13 on MUC5AC gene expression on seven individual donors, as well as Western blotting on cell washings to assess secreted protein. These approaches confirmed that IL-13 treatment caused a significant increase in MUC5AC mRNA and protein production (Fig. 5 *D* and *E*).

A recent study showed that, despite mucus removal, IL-13 treatment had protective effects against SARS-CoV-2 infections (37). Our infections were performed via bolus (200 µL) incubation for 2 h, and this approach was shown to remove the majority of the mucus from the cell surfaces (40). However, to ensure complete removal of mucus, NT and IL-13–treated cells were thoroughly washed with phosphate-buffered saline (PBS; 3 × 1 h) prior to infection and compared with unwashed cells (Fig. 5*F*). In this experiment, IL-13 treatment significantly reduced viral replication, and PBS washes slightly, but not significantly, increased viral replication in both NT and IL-13–treated cells (ΔCt values were +0.69 *P* = 0.16 for NT and +0.97 *P* = 0.33 for IL-13). To further investigate the role played by mucins in the IL-13–induced protection, we knocked out MUC5AC gene expression in the Calu3 lung-derived cell line and showed that IL-13 treatment significantly reduced viral load in these cells as well (*SI Appendix*, Fig. S11). These results indicated that IL-13 had protective effects irrespective of MUC5AC hypersecretion, suggesting that other mechanisms such as alteration in gene expression may be involved. Note that Calu3 cells are rich in goblet cells and devoid of ciliated cells, and express high levels of ACE2 receptor mRNA; however, viral loads were lower in Calu3 than HAE cells, which is consistent with the fact that goblet cells are less competent than ciliated cells at replicating the virus.

In IL-13–treated HAE cells, mucus may further protect the cells against infection, which was tested by comparing bolus versus drop inoculations (*SI Appendix*, Fig. S12). The drop method consisted of applying a small 10-µL inoculum (containing the same number of viral particles as the bolus solution) on the cultures, permitting mucus to remain on the cell surfaces at all times. Drop-infected cells showed additional protection, which was attributable to mucus hyperproduction, since thoroughly washing the cell surfaces of these cells normalized viral load to bolus-infected levels.

### IL-13 Affected Genes Involved in Mucosal Defenses and Viral Pathogenesis.

**Fig. 6.**
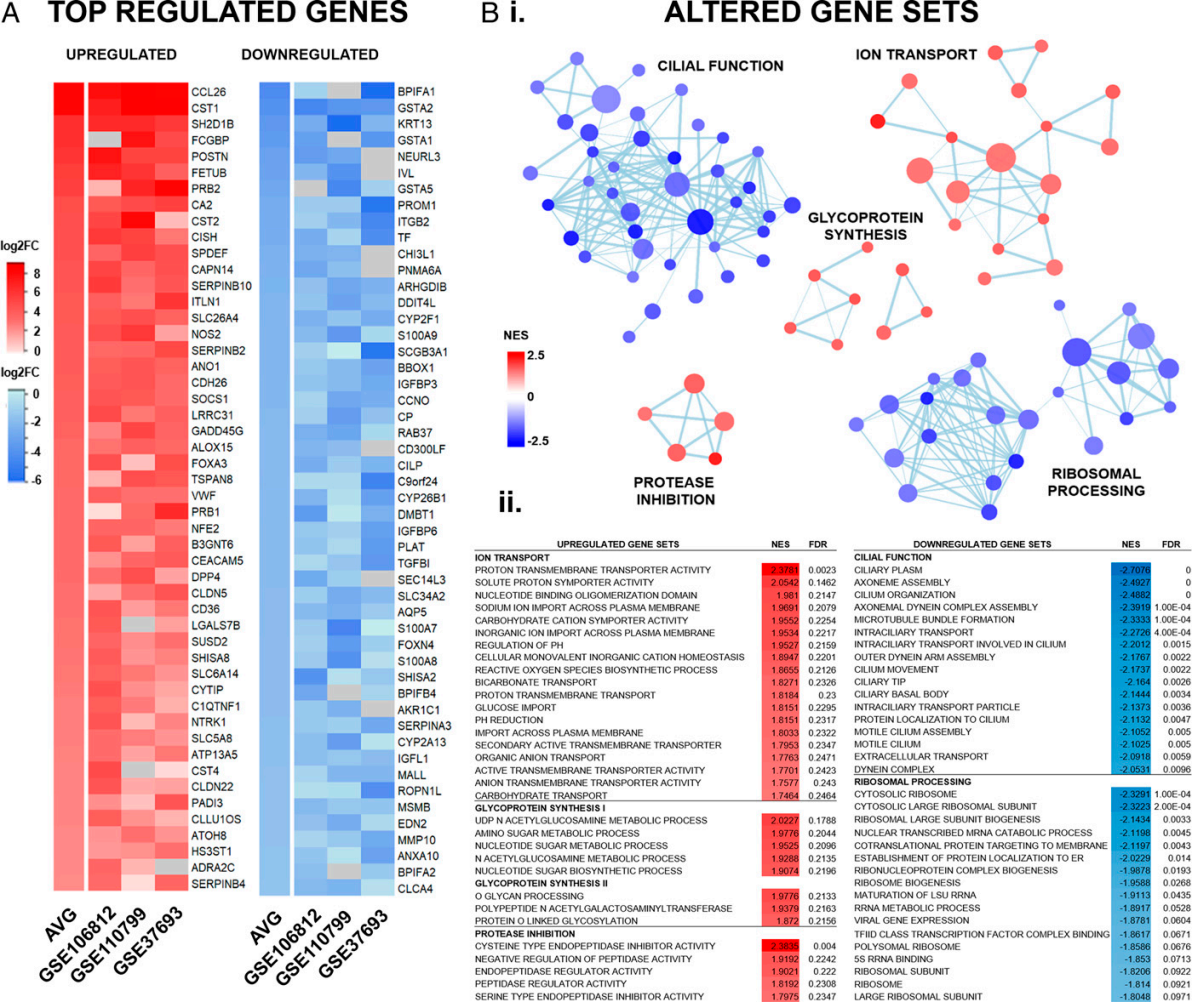
Transcriptome analysis of genes affected by IL-13 treatment in HAE cell cultures. RNA expression data from three independent (GSE106812, GSE110799, and GSE37693) studies of primary HAE cells treated with IL-13. GSE were downloaded from NCBI GEO and analyzed individually and combined (AVG). Raw expression of genes was transformed to log2 fold-change (log2FC) values using GEO2R for comparison across all studies. Gene set enrichment analysis was performed using GO gene sets, and related sets were visualized using Cytoscape. (*A*) Heatmap of the combined top 50 up-regulated (red) and down-regulated (blue) genes across all three studies. Data are shown as log2FC compared to controls. Gray boxes indicate genes not included in the GSE study. (*B*) Top relevant gene sets with significantly altered expression following IL-13 treatment, as shown by network diagram (*i*), accompanied by gene set lists (*ii*). Gene set lists were ranked by the normalized enrichment score (NES) with the false discovery rate q value (FDR) < 0.25.

To investigate changes in the HAE cell transcriptome in response to IL-13, we performed data mining of gene expression datasets (GSE106812, GSE110799, and GSE37693) of primary HAE cells treated with IL-13 available through the National Center for Biotechnology Information (NCBI) GEO repository (41–43). Among the top up-regulated genes were Th2-high asthma biomarkers (CCL26, FCGBP, ITLN1, NOS2), serine and cysteine protease inhibitors (CST1-2/4, SERPINB2/4/10), and secretory transcription factors (SPDEF, FOXA3), while the most down-regulated genes were associated with ciliopathies (CCNO, CFAP161, DNAAF3) or the glutathione S-transferases (GSTA1, GSTA2, GSTA4, GSTA5), of the S100 calcium binding protein family (S100A7-9), the cytochrome P450 monooxygenases (CYP2F1, CYP26B1, CYP2A13), and intracellular vesicle transport genes (RAB37, SEC14L3) (Fig. 6*A*).

In addition to individual genes, we performed a gene set enrichment analysis to detect biological pathways impacted by IL-13 treatment. Several supersets of biologically related gene ontology (GO) processes were elucidated by Cytoscape network analysis (Fig. 6*B* and *SI Appendix*, Fig. S13). The major up-regulated supersets included ion transport (39 sets), glycoprotein synthesis (8 sets), and protease inhibition (5 sets), while gene sets involved in cilia function/ciliogenesis (40 sets) and ribosomal processing (24 sets) were substantially down-regulated. Importantly, IL-13 did not induce expression of interferon-stimulated genes (ISGs), suggesting that the antiviral activity demonstrated occurs via an interferon-independent pathway (*SI Appendix*, Fig. S14). This transcriptome analysis showed that IL-13 improves mucosal defenses by up-regulating mucus and extracellular matrix components along with hydration, and by increasing protease inhibitors with antiviral properties. Simultaneously, IL-13 hindered several mechanisms used for viral replication and spread, including protein synthesis and ciliary activity.

### Effects of IL-13 on Viral Access and Spreading.

**Fig. 7.**
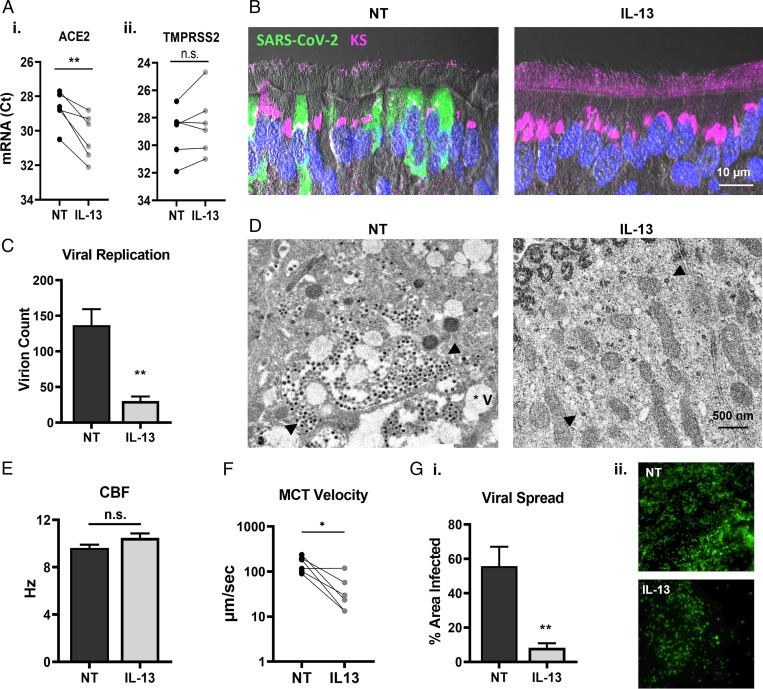
Effects of IL-13 on viral access and spreading. (*A*) Effects of IL-13 on gene expression for viral entry markers in mock cultures, as shown by normalized Ct values for ACE2 (*i*) and TMPRSS2 mRNA (*ii*). (*B*) IHC comparing NT and IL-13–treated cultures at 72 hpi stained with SARS-CoV-2 in green and KS in magenta. Nuclei were stained with DAPI (blue). (*C*) Viral replication established by intracellular virion count per cell via TEM imaging within a 24-h growth cycle. (*D*) Representative TEM images of the cytoplasm of infected ciliated cells at 24 hpi, revealing that the number virions and the extent of intracellular damage were both reduced in IL-13–treated cells. (*E*) CBF measured in NT and IL-13–treated mock cultures. (*F*) MCT velocity rates in paired NT and IL-13–treated mock cultures. (*G*) Effects of IL-13 on viral spread at 96 hpi as determined by the percent area covered with viral signal (green) on whole mounts (*i*), with representative images (*ii*); *n* = 4 inserts per group for two donors. *P < 0.05, **P < 0.01.

We investigated the effects of IL-13 on selected biological processes involved in viral proliferation. In the first part of the study, we established that lower ACE2 mRNA expression correlated with lower viral infection in different donor cells. In Fig. 7*A*, we showed that ACE2, but not TMPRSS2, mRNA expression was down-regulated by IL-13 in all six donors. Since ciliated cells are the primary targets for SARS-CoV-2, the glycocalyx coating of the cilia may play a critical role in governing viral entry. Keratan sulfate (KS), originally identified as the major glycosaminoglycan of the cornea, was shown to be localized to the PCL in the airway epithelium (17). GO analysis revealed that several gene sets involved in KS metabolic processes were up-regulated by IL-13, which correlated with a strong intracellular KS signal emanating from ciliated cells and an increased extracellular KS density coating the cilia (Fig. 7*B*). In addition to the presence of a thicker PCL shield, TEM images following a short viral growth cycle (24 h) revealed that infected cells treated with IL-13 exhibited minimal intracellular damage (even at later time points; *SI Appendix*, Fig. S15), with fewer virions and vacuoles per cell, consistent with a down-regulation of viral replication (Fig. 7 *C* and *D*). Increased mucus production, thicker PCL shield, and down-regulation of multiple genes involved in cilial function in response to IL-13 treatment could alter cilia dynamics and affect viral clearance. We measured the effects of the cytokine on ciliary beat frequency (CBF) and MCT in mock HAE cell cultures and showed no effect on CBF and a moderate but significant decrease in MCT (Fig. 7 *E* and *F*). Diminished MCT velocity correlated with an approximately sevenfold reduced coverage or percent area occupied by infected cells in IL-13–treated cells at 96 hpi, suggesting that the spread or dissemination of viruses was slowed in the cultures (Fig. 7*G*). In the lungs, slower MCT may reduce viral clearance and therefore negatively affect patients with a high-Th2 profile. More work is needed to determine the effects of IL-13 in vivo.

## Discussion

In the past two decades, the acceleration of coronavirus outbreaks reflects an increase in tropism and transmissibility for this large family of viruses (44, 45). Despite belonging to the same beta coronavirus genus as SARS-CoV and MERS, SARS-CoV-2 rapidly emerged as a highly transmissible human pathogen from early stages of infection, suggesting intense viral shedding from patients in a quiescent disease or asymptomatic state (46). Understanding the life cycle of SARS-CoV-2 in airway cells could reveal potential targets to prevent viral spread and advance therapeutic interventions.

Studying viral infections in human tissues is challenging as it typically requires analyses of specimens postmortem, and necropsy tissues are typically collected weeks after the initial infection. Although in vitro models do not fully replicate the whole lung, HAE cell cultures reliably reproduce the pseudostratified epithelium that lines the respiratory tree and exhibit the basic cellular functions of airway cells, including mucus secretion and MCT (47). In the airways, ACE2 is expressed by ciliated and goblet cells (7, 8). However, we demonstrated a marked tropism of SARS-CoV-2 for ciliated cells in our HAE cultures, revealing that only 5% of infected cells were goblet cells. Targeting of ciliated cells by SARS-CoV-2 was associated with rapid viral replication kinetics, with viral titers and viral protein mRNA expression increasing exponentially within hours following infection. Consistent with these data, massive viral shedding was observed by SEM imaging, depicting large clusters of SARS-CoV-2 virions released from the microvilli and membrane protuberances, starting as early as 24 hpi. Studies on norovirus and rotavirus revealed that viruses maintained in clusters exacerbate the spread and severity of disease compared to individual virus particles for equivalent inoculum titers (48). An interesting observation was that infected goblet cells, a cell type that possesses intricate and highly regulated secretory machinery, did not appear to form virion-filled vacuoles, while ciliated cells, a cell type less adapted to apical secretion, presented numerous vacuoles. Further studies, possibly with single-cell genomics and lineage tracing following infection, are warranted to elucidate the relationship between host cell and viral induced secretory mechanisms. In addition, SEM revealed major abnormalities in infected cells that consisted of aberrant cilial organization, membrane enlargement, and pronounced anoikis, starting between 24 and 48 hpi. Disorganized cilia and ciliated cell shedding have been described in other respiratory viral infections and were associated with acquired ciliary dyskinesia and reduced MCT, suggesting that SARS-CoV-2 tropism for ciliated cells and subsequent epithelial damage may affect airway clearance (49). These results are consistent with pathologic observations performed on COVID-19 lung autopsies that showed extensive epithelial damage and cell shedding, resulting in basal cell exposure and airway obstruction.

In virology, the consensus on transmission is based on the notion that viruses operate as independent infectious units. However, viral clustering may significantly facilitate person-to-person transmission, as well as accelerate the ability to colonize different regions of the lungs or the body. TEM imaging confirmed that infected cells contained numerous vacuoles packed with SARS-CoV-2 virions and the fate of shed cells culminated in cell lysis, releasing large numbers of virions from a mobile source. Ciliated cell anoikis combined with a propensity to form virus-laden vacuoles for this cell type may contribute to the elevated transmissibility and virulence of SARS-CoV-2. While intense viral egress was detected as early as 24 hpi in HAE cultures, the onset of clinical symptoms averages around 5 d postexposure with the earlier variants (50). Additional work is needed to determine cell tropism and vacuole formation with the recent Omicron variant. Conceivably, massive viral and cell shedding from the upper respiratory tract play an important role in the early transmission of SARS-CoV-2 from presymptomatic or asymptomatic individuals. Some of the key adaptation mechanisms from SARS-CoV-2 to ensure rapid, efficient spread may have emerged from viral clustering combined with the trafficking of large vacuoles containing tightly packed viruses. The effectiveness of mask wearing corroborates this notion (51, 52).

To prevent the spread of infection, the lungs typically respond to pathogen invasion by increasing mucus production, with MUC5AC being the dominant mucin for pathogen trapping (21–23). The rapid replication kinetics of SARS-CoV-2 may not provide sufficient time for goblet cell hyperplasia, which might explain how the lungs become overwhelmed in a number of infected individuals. In naïve HAE cells, we showed that MUC5AC intracellular storage was depleted within 2 d to 3 d following infection, resulting in extracellular MUC5AC circumjacent shed viruses and sloughed cells. In the later time points, goblet cells failed to replenish mucin storage, suggesting that SARS-CoV-2 fast replication overwhelmed naïve mucin-secreting cells.

MUC5AC is overproduced in COPD and asthmatic patients (22, 28); however, the majority of these patients are at higher risk of complications from COVID-19 (24, 25), suggesting that MUC5AC alone cannot block SARS-CoV-2 infection. However, patients with Th2-high asthma endotype characterized by high levels of IL-13 have recently emerged as less susceptible to severe COVID-19 (26, 28, 36). We demonstrated that IL-13 administration in HAE cells markedly reduced SARS-CoV-2 titers, viral gene expression, and epithelial damage, and affected the number of infected cells. In in vitro models, the absence of innate immune response creates limitations to fully understanding the role of IL-13 in the human lungs. Although mice infected with a mouse-adapted SARS-CoV-2 revealed a different cellular tropism (53), in vivo experiments on relevant mouse models (e.g., house dust mite, IL-13R KO, MUC5AC KO, and MUC5AC-overexpressing mice) should be conducted to gain better insights into the role of Th2 cytokines and mucus in SARS-CoV-2 infection. Although MUC5AC expression was up-regulated and viral access was reduced in IL-13–treated cells, IL-13 treatment provided additional protective effects. Despite removal of mucus via HAE cell washings or the use of MUC5AC KO Calu3 cells, viral loads remained significantly lower in IL-13–treated cells, indicating that other cellular mechanisms were involved. Data mining of bulk RNA-sequencing experiments highlighted several biologic processes affected by IL-13 that could affect viral proliferation, including viral entry, replication, and spread. Additional analyses showed that the effects of the Th2 cytokine were not related to the typical ISGs that have been shown to inhibit SARS-CoV-2 infections (54). To validate some of these findings, we showed that ACE2 mRNA expression was down-regulated by IL-13 and that KS coating the cilia formed a thicker layer in treated cells, both decreasing viral access to the targeted cell type. In addition, several genes involved in ribosomal function were down-regulated by IL-13, which correlated with slower replication kinetics, fewer infected cells, fewer virions per infected cells, and an impaired ability to form vacuoles. Reduced epithelial damage coincided with reduced anoikis and limited spread of infection in IL-13–treated HAE cell cultures. Individually or in combination, these biological processes warrant further investigations to elucidate innate mechanisms protecting against SARS-CoV-2 infection.

In conclusion, SARS-CoV-2 infection of HAE cell cultures is an important model to investigate the replication and spreading mechanisms used by SARS-CoV-2 in human airway epithelia. Tropism for ciliated cells was associated with severe cytopathic effects that caused cell shedding and membrane disruption, ensuring the rapid release of large numbers of virions. This process was observed shortly after inoculation and may play an important role in early transmission. Treatment with IL-13 significantly diminished viral shedding and cellular damage by affecting viral entry and replication, which appear to play an important role in the protective effects against SARS-CoV-2 infection.

## Materials and Methods

For complete materials and methods, see *SI Appendix*. Primary HAE cells were harvested from donor lungs, grown on air–liquid interface (ALI), and allowed to differentiate 5 wk before experimentation (47). Cells treated with IL-13 received 1 ng/mL of IL-13 daily in the basolateral media starting 3 d prior to infection (34). Calu3 cells were grown on ALI, and CRISPR-Cas9 was used to knock out MUC5AC, which was confirmed by Western blotting. Cell cultures were infected apically with SARS-CoV-2 at a multiplicity of infection (MOI) of 0.5 and processed at various time points. Viral titers were obtained by measuring plaque-forming units (PFU) per milliliter on Vero-E6 cells (10). Gene expression was assessed using qRT-PCR (34). RNA-ISH was performed on formalin-fixed paraffin-embedded HAE sections using the RNAscope Reagent Kits (10). HAE sections were also stained with H&E, AB-PAS, and IHC with corresponding antibodies. Apoptotic cells were detected using the Click-iT-Plus TUNEL Assay. Fluorescence was detected with an Olympus VS120 virtual scanning microscope and light microscopy using a Nikon e200 microscope. SEM samples were imaged using a Supra 25 field emission scanning electron microscope (11, 12). TEM samples were imaged using a JEOL JEM-1230 transmission electron microscope or an FEI Tecnai 12 transmission electron microscope operating at 120 kV (11, 23). Relative abundance of MUC5AC was analyzed by mucin Western blotting (55). Gene expression data were obtained from NCBI GEO and processed using R. CBF was determined by taking phase contrast videos of the field of view and performing Fourier spectral analysis (37). MCT velocity was obtained by tracking hardened red blood cells. Viral spread was quantified as the percent area of SARS-CoV-2 signal on whole-mount immunostained inserts. Cell dimensions were quantified using ImageJ. Cell and virion counts were obtained using a manual cell counter in a blinded manner. Statistical analysis was performed using GraphPad Prism with significance assessed at *P* < 0.05.

## Supplementary Material

Supplementary File

## Data Availability

GEO datasets used in this study (GSE106812, GSE110799, GSE37693) are available through NCBI. All other study data are included in the article and/or *SI Appendix*.
