# Supplementary material for: SARS-CoV-2 infection of airway cells causes intense viral and cell shedding, two spreading mechanisms affected by IL-13
Source: Proc Natl Acad Sci U S A. 2022 Mar 30;119(16):e2119680119. doi: 10.1073/pnas.2119680119 (PMC9169748; doi:10.1073/pnas.2119680119)
Supplement: Supplementary File [file pnas.2119680119.sapp.pdf]

Supplemental Information

SARS-CoV-2 Infection of Airway Cells Causes Intense Viral and Cell Shedding, two Spreading Mechanisms Affected by IL-13

Cameron B. Morrison[1]*, Caitlin E. Edwards[2]*, Kendall M. Shaffer[1], Kenza C. Araba[1], Jason A. Wykoff[1], Danielle R. Williams[3], Takanori Asakura[1], Hong Dang[1], Lisa C. Morton[1], Rodney C. Gilmore[1], Wanda K. O'Neal[1], Richard C. Boucher[1], Ralph S. Baric[2,3] and *Camille Ehre[1,4]

Affiliations:

[1]Marsico Lung Institute, The University of North Carolina at Chapel Hill, Chapel Hill, NC, 27599, USA

[2]Department of Epidemiology, The University of North Carolina at Chapel Hill, Chapel Hill, NC, 27599, USA

[3]Department of Microbiology and Immunology, The University of North Carolina at Chapel Hill, Chapel Hill, NC, 27599, USA

[4]Department of Pediatrics/Pediatric Pulmonology, The University of North Carolina at Chapel Hill, Chapel Hill, NC, 27599, USA

* Both authors participated equally to the work

Correspondence should be addressed to: camille_ehre@med.unc.edu

Materials and Methods:

Primary HAE cell cultures: Primary human airway epithelial (HAE) cells were harvested from transplanted lungs or post-mortem and de-identified for further procedure (1). Procedures involving human cells were approved by the Institutional Review Board at the University of North Carolina Chapel Hill (IRB # 03-1396, 12-2602). Cells were grown on 12 mm Transwell inserts coated with 7.5µg/cm^2 type IV collagen (Sigma C-7521) with PneumaCult-Ex Plus media (StemCell Technologies, Vancouver, Canada) for 3-5 days. Once confluent, cells were switched to air-liquid interface (ALI) and differentiated with PneumaCult-ALI media basolaterally. Media was changed every other day for five weeks prior to infection.

IL-13 treatment *in vitro*: Three days prior to infection, HAE cultures were divided into two groups, the PBS control or non-treated (NT) and the IL-13-treated (IL-13) groups, previously described in Chen et al. (2). HAE cells were treated daily via the basolateral media with PBS or 1ng/ml IL-13 (Preprotech, USA). IL-13 treatment was maintained throughout the course of SARS-CoV-2 infections.

Infection with SARS-CoV-2: HAE cultures were infected with 200ul of SARS-CoV-2 D614G diluted in PBS to a multiplicity of infection (MOI) of 0.5 applied to the apical surface. Cultures were allowed to incubate at 37°C for 90 minutes. Following incubation, virus inoculum was removed, and cultures were washed on the apical surface 3X with 500ul PBS. Cultures were returned to 37°C and allowed to incubate. HAE cells were processed a various time points (24, 48, 72, and 96 hours post infection or hpi) for microscopy, washes or mRNA collection (see below for details).

Viral Titers: Mock and infected cultures were washed with 200µL PBS for 10 min and titered via plaque assay as described by Hou YJ 2020 (3). Briefly, washings were serially diluted 10^{-1} to 10^{-6}, applied to Vero-E6 cell monolayers and overlayed with agarose. Plaques were visualized with neutral red stain 3 days later and counted as plaque-forming units per ml (PFU/ml).

Gene expression by qPCR: HAE cultures were lysed in TRIzol Reagent (Invitrogen, Carlsbad, CA), processed for total mRNA extraction, followed by cDNA reverse transcription, and quantitative PCR as described by Chen et al. (2). In brief, total RNA was purified via the Direct-Zol RNA miniprep kit (Zymo Research, Irvine, CA). RNA was reverse transcribed to cDNA and qPCR was performed using TaqMan probes with SsoAdvanced Universal Probes Supermix on QuantStudio6 Flex Real-Time PCR System (Applied Biosystems, Foster City, CA) for MUC5AC, ACE2, and TMPRSS2. GAPDH was used as house-keeping gene for normalization. For the nucleocapside gene expression, qPCR was performed using the N1 and N2 primer sets from the 2019-nCoV CDC EUA kit (IDT, Cat# 10006770) according to the CDC protocol (4). The standard curves of N1 and N2 primer sets were generated by 2019-nCoV N positive control plasmid (IDT, cat# 10006625). For additional information, see https://www.fda.gov/media/134922/download. Results are shown as fold change ($2^{-\Delta\Delta Cp}$) in mRNA expression or relative changes in cross-point (Cp) values.

Mucin Western blotting: Relative abundance of MUC5AC was analyzed by mucin Western blotting as previously described by Ramsey et al (5). Mock and infected HAE cultures were washed with 200µL PBS for 10 min. To inactivate SARS-CoV-2, washings were diluted 1:1 in 8M urea before removal from BSL3 facility. Cell washing samples (40 µl) were separated by electrophoresis using a 0.8% agarose gel (80V) for 60 min. Samples were then vacuum transferred to a 0.45µm nitrocellulose membrane. Following transfer, membranes were blocked for 1h at RT and probed with 45M1 mouse-anti-MUC5AC (Invitrogen MA5-12178, Invitrogen, Carlsbad, CA) overnight at 4°C. Membranes were rinsed in PBS and then probed for secondary detection with 1µg/mL donkey-anti-mouse (Li-Cor 926-68022, Li-Cor, Lincoln, NE) for 1h at RT. Lane signal intensity was measured using the Li-Cor Odyssey software.

RNA *In situ* hybridization (RNA-ISH): RNA-ISH was performed on tissue sections that were deparaffinized with xylene (2 changes 3 x 5 min) and 100% ethanol (2 changes 3 x 1 min), and then incubated with hydrogen peroxide for 10 min followed by target retrieval solution for 15 min, and

incubation with Protease Plus (Advanced Cell Diagnostics) for 15 min at 40°C. *In situ* RNA detection was performed using the RNAscope® 2.5 HD Duplex Reagent Kit Cat# 322430 from Advanced Cell Diagnostics Inc. (ACD), Hayward, CA. The probes used were human virus RNAscope® Probe- V-nCoV-2019-S Cat No. 848561-C1 (Channel 1), RNAscope® Probe- Hs-ACE2-C2 Cat No. 848151-C2 (Channel 1), RNAscope® 2.5 Duplex Positive Control Probe (Hs) PPIB-C1/POLR2A-C2 Cat#321641 and negative control probe was DapB (4-hydroxy-tetrahydrodipicolinate reductase from *Bacillus subtilis*) Cat# 310043. Slides were then hybridized with the probes in the HybEZ oven (ACD), at 40°C for 2 hours.). After hybridization, slides were subjected to signal amplification according to the manufacturer's instructions. Hybridization signals were detected using a mixture of solutions A and B (1:60) in RNAscope® 2.5 HD Duplex Assay. After counterstaining with 50% hematoxylin, slides were dried in a 60°C dry oven for 30 minutes and mounted with VectaMount (ACD). Note, the RNAscope® 2.5 HD Duplex kit system allows for detection of variations in mRNA abundance of individual genes labeled in cyan for Channel 1 (SARS-CoV-2) and magenta for Channel 2 (ACE2). The signal intensity obtained from each probe is probe-specific. ACD made the probes and reagent kits for RNA *in situ* hybridization based on proprietary RNAscope technology. The integrated probe design with signal amplification and detection are used to achieve single-molecule detection. Images were acquired with an Eclipse e200 brightfield microscope (Nikon, Tokyo, Japan) at 100X with oil objective.

Scanning electron microscopy: As previously published in Ehre C. et al. (6), cultures were gently submerged in 2.5% glutaraldehyde in 0.1M sodium cacodylate buffer (Electron Microscopy Sciences, Hatfield, PA) overnight at 4°C and rinsed twice with 4% PFA. Samples were rinsed with 0.1M sodium cacodylate buffer to remove any fixative before osmication and dehydration. For osmium tetroxide fixation (OTO), samples were gently submerged in a solution of 1% OsO4 (Electron Microscopy Sciences, Hartfield, PA) in deionized water for 1h. Samples were washed 5X for 5 min with deionized water to remove the osmium solution before submerging in filtered 1% tannic acid (Alfa Aesar, Haverhill, MA, Cat #A1702230) in deionized water for 30 min. Samples were washed again with

deionized water to remove the tannic acid solution and then submerged in 1% OsO4 for 15 min. Fixed cultures were then rinsed 3X for 5 min with diH_2O. Subsequently, samples were dehydrated in 6X 10 min baths of increasing concentrations of ethanol (30%, 50%, 75%, 95%, 100%, 100%) and dried using a Samdri-795 liquid CO2 critical point drier (Tousimis Research Corp, Rockville, MD). Following drying, samples were mounted on aluminum planchets with carbon adhesive tabs (cell inserts) and coated with 5 nm gold palladium alloy (60Au:40Pd) using a Ted Pella 208HR sputter coater (Ted Pella Inc, Redding, CA). Samples were imaged using a Supra 25 field emission scanning electron microscope operating at 5kV with a working distance of 5 mm and 30 µm aperture (Carl Zeiss Microscopy, Peabody, MA). Adobe Photoshop (San Jose, CA) was used to colorize SEM images.

Transmission Electron Microscopy: Cultures were gently submerged in 4% paraformaldehyde and 2.5% glutaraldehyde in 0.1 M sodium cacodylate (Electron Microscopy Sciences, Hatfield, PA), as described in Ehre et al. (7) and Hou Et al. (8). Samples were rinsed with sodium cacodylate buffer and were post-fixed for 1h in potassium-ferrocyanide reduced osmium (1% osmium tetroxide/1.25% potassium ferrocyanide/0.1 sodium cacodylate buffer). After rinsing in deionized water, the cells were dehydrated using increasing concentrations of ethanol (30%, 50%, 75%, 90%, 100%, 100%, 10 minutes each) followed by infiltration and embedment in Polybed 812 epoxy resin (Polysciences, Inc.,Warrington, PA). The cells/transwell inserts were sectioned perpendicular to the substrate at 70nm using a diamond knife and Leica UCT ultramicrotome. Ultrathin sections were collected on 200 mesh copper grids and stained with 4% aqueous uranyl acetate for 15 minutes, followed by Reynolds' lead citrate for 7 minutes. Samples were observed using a JEOL JEM-1230 transmission electron microscope operating at 80kV (JEOL USA, Peabody, MA) or an FEI Tecnai 12 transmission electron microscope operating at 120kV (Thermo Fisher Scientific, Hillsboro, Oregon, United States) and images were taken using a Gatan Orius SC1000 CCD camera with Gatan Microscopy Suite 3.0 software (Gatan, Inc., Pleasanton, CA).

Immunohistochemistry: Primary HAE cultures fixed in 4% PFA were embedded in paraffin and prepared as histological sections or used for whole-mount staining. Paraffin-imbedded sections were deparaffinized and stained with hematoxylin and eosin (H&E) or processed for immunohistochemistry (IHC), as described in Ehre (7). Briefly, IHC unstained sections underwent epitope retrieval using heated 1X citrate buffer in deionized water, and whole-mount cultures in transwells were permeabilized with 0.2% Triton X-100 for 30 minutes at room temperature. Both unstained sections and whole-mount inserts were blocked for 1h in 3% Bovine Serum Albumin (BSA) solution. For unstained slides, primary antibodies: rat anti-X-tubulin (Millipore Sigma MAB1864), mouse anti-MUC5AC 45M1 (Invitrogen MA5-12178) and rabbit anti-SARS-CoV-2 nucleoprotein (Abclonal A18797) were co-applied overnight at 4°C, followed by secondary antibodies (anti-rat AlexaFluor 594, anti-mouse AlexaFluor 647 and anti-rabbit AlexaFluor 488) incubation for 1h at room temperature. For keratan sulfate-stained slides, the primary antibody: mouse monoclonal anti-keratan sulfate clone 5D4 (Amsbio 270427-CS) was co-applied with the Abclonal SARS-CoV-2 antibody overnight at 4°C followed by secondary antibodies (anti-mouse AlexaFluor 488 and anti-rabbit AlexaFluor 594) for 1h at room temperature. Slides were incubated with DAPI (Invitrogen 2031179) for 5 min at room temperature. Whole mount inserts were stained with rabbit anti-SARS-CoV-2 nucleoprotein (Abclonal A18787) and its secondary antibody, anti-rabbit AlexaFluor 488, only. Slides were mounted using FluorSave reagent, and fluorescence was detected with a VS120 virtual scanning microscope (Olympus Microscopy, Shinjuku, Japan) or a Leica Stellaris5 confocal microscope (Leica Microsystems, Wetzlar, Germany).

TUNEL Assay: Apoptotic cells in SARS-CoV-2-infected primary HAE cultures were detected using the Click-iT™ Plus TUNEL Assay (Invitrogen, Carlsbad, California, Cat #C10618). Following primary and secondary antibody labeling per IHC protocol (see above), TUNEL staining was performed according to the manufacturer's protocol. Briefly, deparaffinized and IHC stained sections were fixed with 4% PFA and permeabilized by Proteinase K treatment. Slides were then incubated at 37°C with

a TdT reaction mixture (Tdt reaction buffer, EdUTP, & TdT enzyme) for 10 minutes. Finally, slides were incubated with the Click-iT™ Plus TUNEL reaction mixture containing the Click-iT™ Plus TUNEL Supermix (1X Click-iT™ Plus TUNEL Reaction Buffer, Copper Protectant, and Alexa Fluor 594 picolyl azide) and 10X Click-iT™ Plus TUNEL Reaction buffer at 37°C for 30 minutes. A five-minute DAPI incubation was performed, followed by PBS and DI H_2O washes. The sections were mounted using FluorSave Reagent (Millipore Sigma 345789) and imaged using a VS120 virtual scanning microscope (Olympus Microscopy, Shinjuku, Japan).

Cell/virion counts and measurements: To measure the width of shed cells, three measurements per cell were performed and averaged using the ImageJ software. The width of 5 and 20 shed cells were measured for the mock and the infected cultures, respectively. For shed cell count, H&E slides were used to count cells fully detached from the epithelium along the entire length of 4 sections/inserts for each group and each time point using the Eclipse e200 brightfield microscope (Nikon, Tokyo, Japan). For SARS-CoV-2-positive cell count, IHC stained slides were used to count individual cells fully attached to the epithelium (i.e., excluding shed cells) that were labeled with the rabbit anti-SARS-CoV-2 nucleoprotein (Abclonal A18797) antibody. Similar to shed cell count, SARS-CoV-2-positive cells were counted along the entire length of 4 sections for each group and each time point using the VS120 virtual scanning microscope (Olympus Microscopy, Shinjuku, Japan). For RNA-ISH analysis, ACE2-positive cell percentage were counted by overlapping consecutive sections stained with H&E (to identify nuclei and ciliated cells), AB-PAS (to identify goblet cells) and RNA-ISH (to identify ACE2+ cells) images. Nuclei count was used to determine the total number of apical cells. Apical cells were determined as cells in direct contact with the apical surfaces of the cultures. For intracellular virion count, TEM images (20 KX magnification) were acquired at 24 hpi focusing on the region between the nucleus and cilia basal bodies of infected cells. Virion count was performed blinded using a cell counter, later analyzed per group (NT vs IL-13) and presented as number of detectable virions per cell.

Generation of Calu3 cells deficient for MUC5AC. CRIPR/Cas9-delivering lentiviruses were used to introduce the Cas9 protein and the single guide RNA (sgRNA) into Calu3 cells to target MUC5AC gene. Three separate MUC5AC-guide RNA sequences were utilized to transfect Calu3 cells. Puromycin was used for selection for >1 week. Ten single clones were screened for complete loss of MUC5AC signal, while these clones remained positive for MUC5B signal, indicating specific mucin targeting. Western blotting were performed on cell washings to detect loss of MUC5AC signal using the mouse monoclonal anti-MUC5AC (45M1) and a rabbit polyclonal anti-MUC5B antibody (UNC414 Ehre lab).

IL-13 HAE Gene Expression Data Mining: Gene expression data was obtained from the NCBI Gene Expression Omnibus (GEO) using the search terms: *("interleukin-13"[MeSH Terms] OR IL-13[All Fields]) AND ("Homo sapiens"[Organism] AND "Expression profiling by array"[Filter]) AND "airway"[All Fields].* 12 datasets were found, of which 3 studies both utilized primary human airway epithelial cell models and treated with IL-13 for >72h (GSE106812, GSE110799, & GSE37693). GEO2R (NCBI, Bethesda, MD) was used to obtain descriptive statistics and match probes to gene identifiers for each dataset. Log2 fold-change (log2FC) values for each gene were matched between datasets, filtered for genes present in at least 2/3 studies, and the average log2FC for all studies was used to obtain the top 50 up- and downregulated genes in all datasets. Heat maps for top up- and downregulated genes were generated using the gplots heatmap.2 function (Warnes et al, 2020) in R (R Core Team, 2021).

IL-13 Gene Set Data Mining: Processed data mentioned above was utilized for gene set analysis. A weighted rank-ordered gene list of all 3 studies was compiled using the formula:

$$\frac{\left(logFC_{(1)} \times (1 - p_{adj_{(1)}})\right) + \left(logFC_{(2)} \times (1 - p_{adj_{(2)}})\right) + \left(logFC_{(3)} \times (1 - p_{adj_{(3)}})\right)}{(1 - p_{adj_{(1)}}) + (1 - p_{adj_{(2)}}) + (1 - p_{adj_{(3)}})}$$

The ranked list was run through GSEA v4.1 (UCSD/Broad Institute) (PMID: 16199517, PMID: 12808457) with 10,000 permutations, gene set size 10≤500, and Gene Ontology database v7.4 (Gene Ontology Consortium) (9, 10) to generate differentially expressed gene sets. Gene set network

visualization was performed using Cytoscape v3.8 (Cytoscape Consortium, San Diego, CA) with a false discovery rate q-value <0.25 and edge similarity threshold of >0.5 (11).

Interferon-Stimulated Genes Data Mining: Two lists of interferon-stimulated genes (ISGs) were obtained from Menachery et al. (12) and Schoggins and Rice (13). Gene expression data from GSE19182 (14) was used to generate type 1 and 2 ISG profiles and data from GSE153428 (15) was used to generate a type 3 ISG profile for comparison to IL-13 datasets. Data filtering and heatmap generation were performed as mentioned above.

Ciliary beat frequency (CBF): Cilia beat frequency (CBF) was determined by taking phase contrast videos of the field of view and performing Fourier spectral analysis on each pixel as a function of time. Twenty-second videos were recorded at 60 frames/sec using a FLIR Grasshopper 3 camera and a 40x objective. Videos were imported into MATLAB, where each pixel was stored as an array of grayscale values versus time. Each array was windowed and then transformed via fast Fourier transform. The dominant peak in the power spectrum was selected and checked against the next highest peaks (2.5 standard deviations away in power) to determine each pixel's light intensity fluctuation frequency and to make sure that the signal was distinct from the noise. Frequencies were reported for each pixel, and the average of all the pixels with nonzero frequencies was used to determine the CBF for a field of view (16).

Mucociliary transport velocity measurements (MCT Velocity): Fluorescent carboxylated beads can bind to the cilia when nebulized directly onto the cells. Hence, for in situ mucus transport measurements, we used red blood cells (RBC) since their surface chemistry is designed to not interfere with mucus transport. To prevent coagulation and cell lysis, RBC collected from laboratory samples were hardened by exposure to 0.08% glutaraldehyde, centrifuged 500xg for 10 min and resuspended in PBS. To measure mucus velocity rates, 2µl of hardened RBC solution were added apically to the center of the cell cultures. RBC were allowed to disperse for 15 min before imaging. Following RBC dispersion, four specific regions (center and three edges) of the inserts were imaged

for 30 sec with a dissecting microscope (SPZV-50E, AVEN Inc.) at 1.5X magnification and 60 frames/s (see videos). On average, 12 RBC streaks (spreading from center to edge) were tracked per insert and the distance travelled was calculated using ImageJ (ImageJ, National Institutes of Health, Bethesda, Maryland, USA). Mucus velocity rate was determined in μm/sec for 4-5 inserts with and without IL-13 treatment.

Viral Spread Measurements: Viral spread was quantified as the percent area of SARS-CoV-2 signal on whole-mount immunostained inserts. Representative images of each infected insert were acquired via a Stellaris5 confocal microscope (Leica Microsystems, Wetzlar, Germany) and the percent area of SARS-CoV-2 fluorescent signal was measured with ImageJ (ImageJ, National Institutes of Health, Bethesda, Maryland, USA).

Statistical analysis: Statistical analysis was performed using GraphPad Prism (GraphPad Software, San Diego, CA). Comparisons between two groups was performed using paired t-test, multiple t-test or independent samples t-test with or without log transformation depending on experimental setting. Comparisons between multiple groups was also performed using a two-way ANOVA followed by *post-hoc* comparisons using Tukey's corrected multiple comparisons test. Significance was assessed at P<0.05. Values in graphs are shown as ±SD.

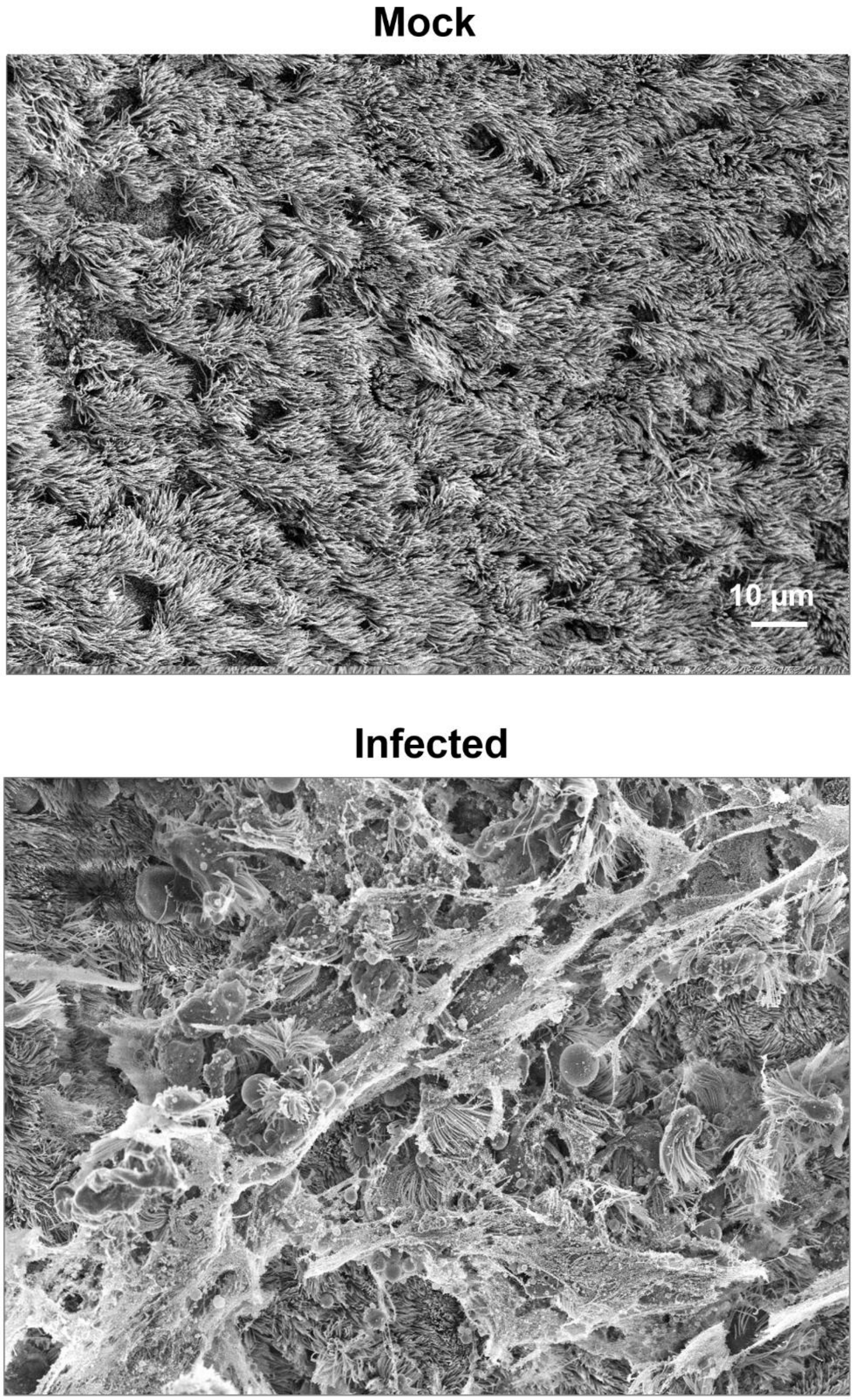

Figure S1. Original SEM micrographs of mock and SARS-CoV-2 infected HAE cultures shown in Figure 1. En face views of a mock HAE culture (top) displaying healthy ciliated cells with traces of mucus and a SARS-CoV-2 infected culture (bottom) exhibiting mucus sheets surrounding ciliated cells in the process of detaching from the cell surfaces.

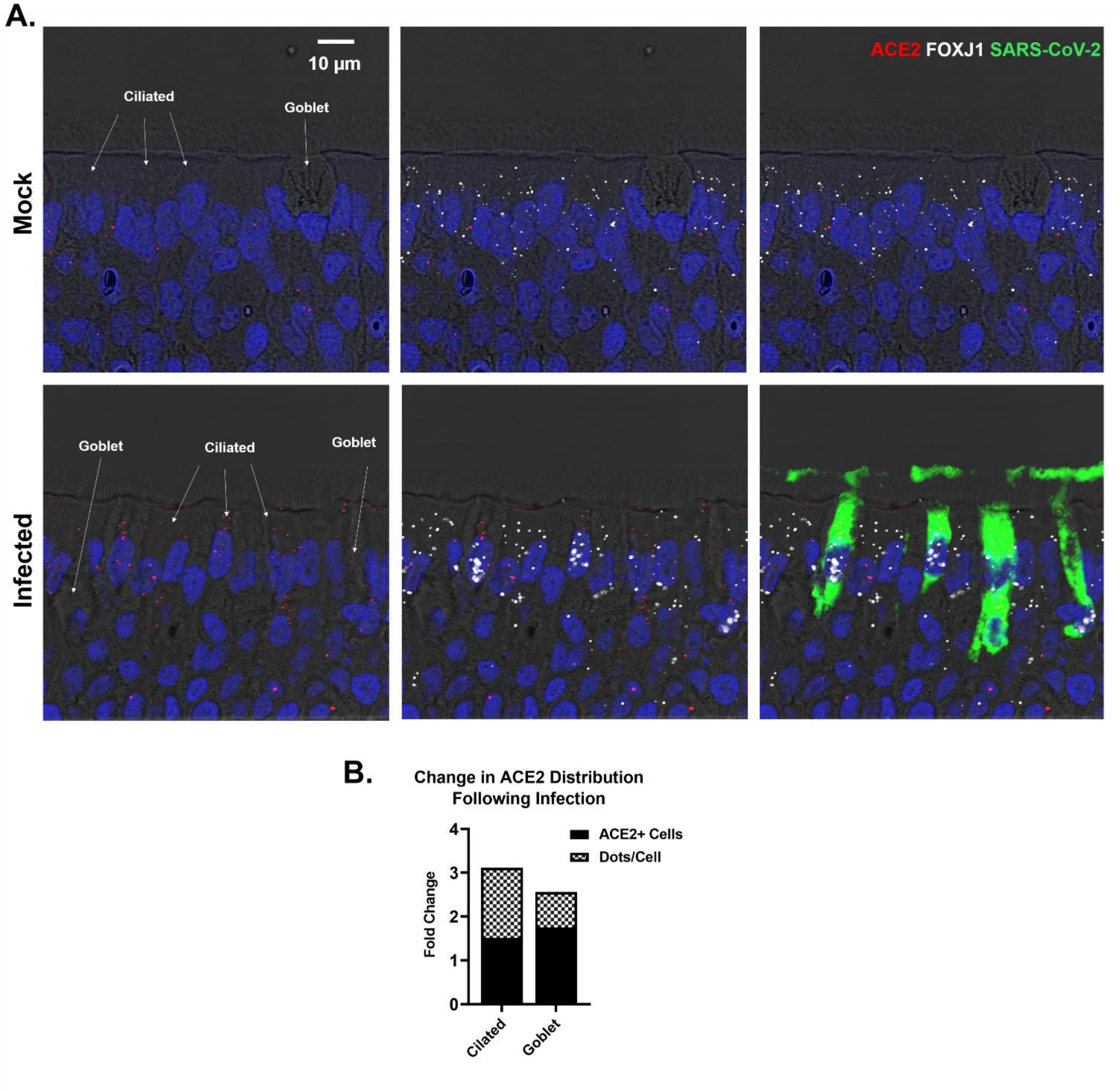

Figure S2. ACE2 distribution on mock and infected HAE cells by Fluorescent RNA-ISH. A.) Representative RNA-ISH images of infected HAE cell cultures at 48 hpi labeled with ACE2 (red), FOXJ1 (white), and SARS-CoV-2 (green) mRNA probe. From left to right, images show the consecutive overlay of DIC and DAPI with the following probes: ACE2, FOXJ1, SARS-CoV-2. A few ciliated and goblet cells were annotated to facilitate visualization. **B.)** Fold change in ACE2 distribution following infection as shown for individual cell type (ciliated and goblet cells), as well as for the number of dots per cell.

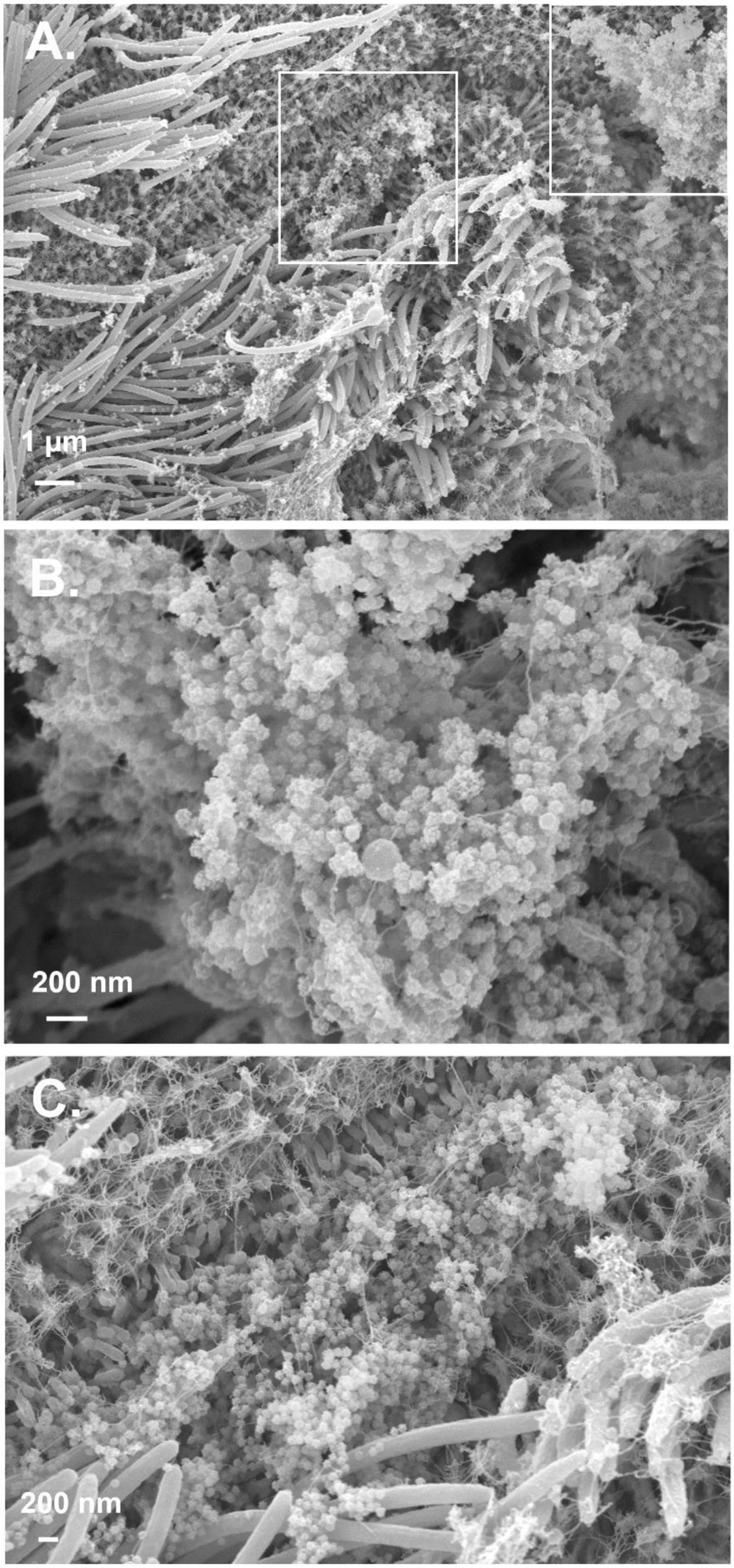

Figure S3. SEM images of SARS-CoV-2 infected HAE cells illustrating extensive viral shedding from a single ciliated cell. **A.)** Low magnification image of an infected ciliated cell that appears slightly sunken and is covered by a thin mucus sheet. White boxes indicate the areas imaged at high resolution in other panels. **B.)** High resolution image of a cluster of virions adjacent to the infected cell. **C.)** Viral shedding across the microvilli or pericellular space. Scale bar is indicated on each micrograph.

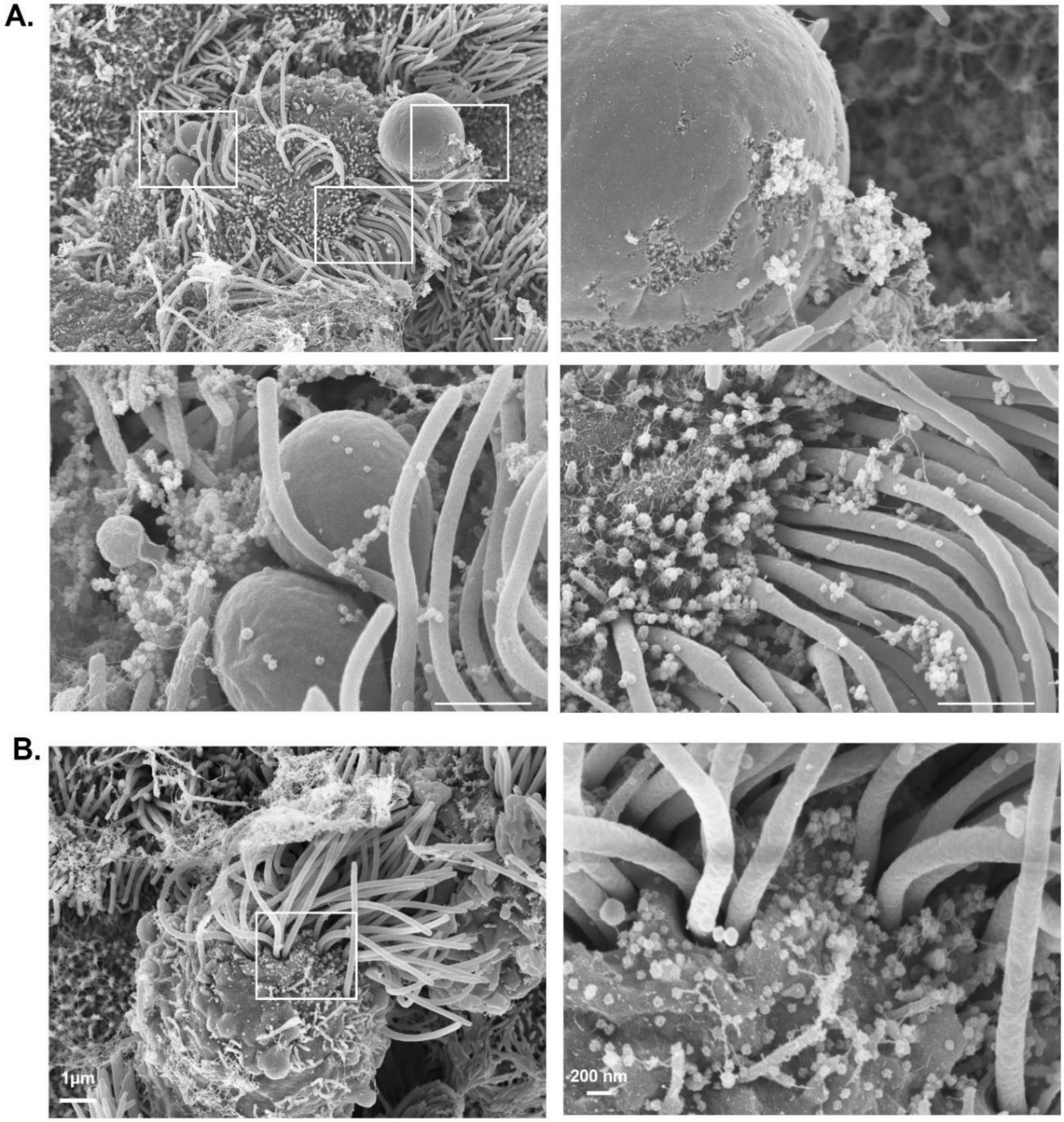

Figure S4. SEM images of infected ciliated cells illustrating cilial shaft disorganization and cell damage caused by SARS-CoV-2 replication. A.) Images of an infected ciliated cell that presented multiple cell protuberances surrounded by a thin mucus sheet. White boxes indicate the areas imaged at high resolution for each individual panel, depicting clusters of virions released from a membrane protuberance bursting open, as well as viral shedding between the cilial shafts and the microvilli. Scale bar is 1µm. **B.)** Additional SEM images of a fully detached ciliated cell exhibiting viral shedding. High magnification image confirms the presence of SARS-CoV-2 virions on the cell surface. Scale bar is indicated on each micrograph.

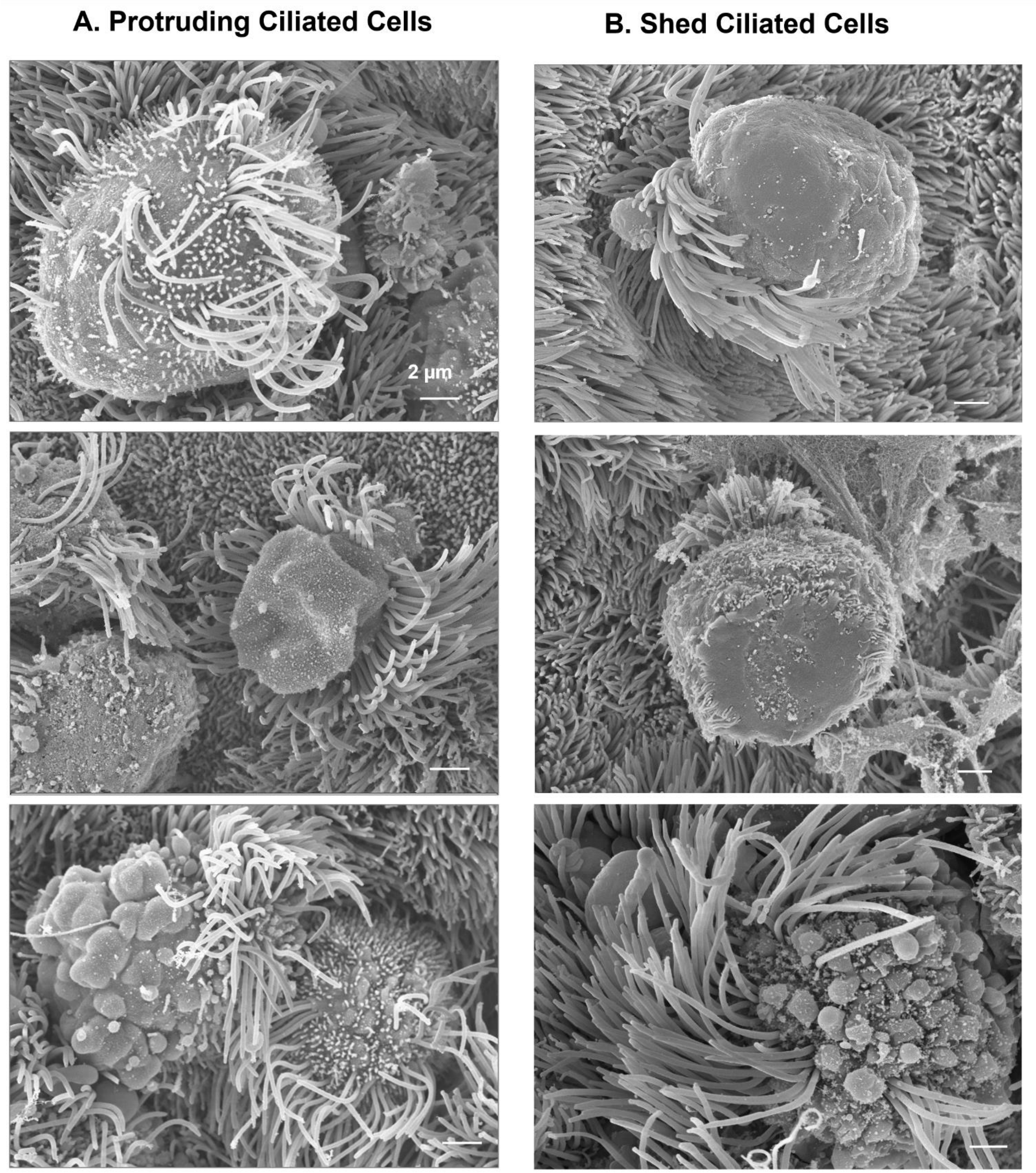

Figure S5. Additional SEM images of SARS-CoV-2 infected HAE cells depicting cell enlargement, protrusion, and detachment from the cell surfaces from three different donors. A.) SEM images of cells protruding outwardly frequently observed in infected cultures. **B.)** Fully detached ciliated cells described herein as a hallmark of SARS-CoV-2 infected airway cultures, revealing the extent of infected cell engorgement. Scale bars are 2 µm.

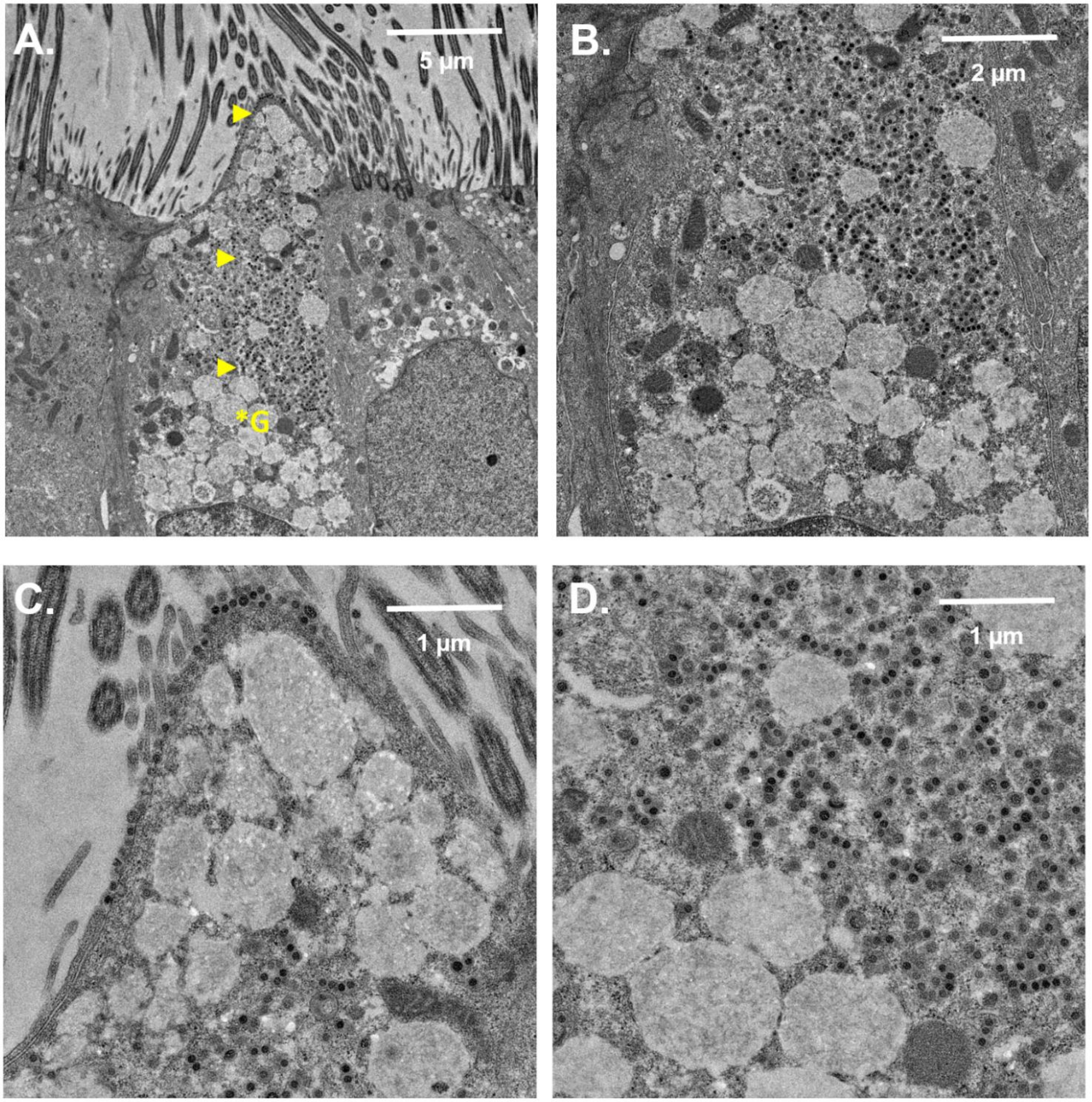

Figure S6. Transmission electron microscopy images of a sporadic SARS-CoV-2 infected goblet cell. HAE cells were infected with SARS-CoV-2 (MOI of 0.5) and processed for TEM at 96 hpi. **A.)** Low magnification TEM images of an infected goblet cell (center) surrounded by infected ciliated cells. In contrast with ciliated cells, no vacuole was observed in infected goblet cells. Annotations indicate *G: goblet cell, ►: SARS-CoV-2 virions. Goblet cell infection was rare (1 goblet vs 19 ciliated cells were infected in this section). **B.)** Mid magnification image showing the presence of large mucin granules (low contrast) and intracellular virions (high contrast) in the same cell. **C.)** High magnification image showing the apical region of the infected goblet cell coated with virions. **D.)** High magnification image of the cell cytoplasm showing numerous individual virions (i.e., not in vacuoles) interspersed between mucin granules. Scale bars are indicated on each micrograph.

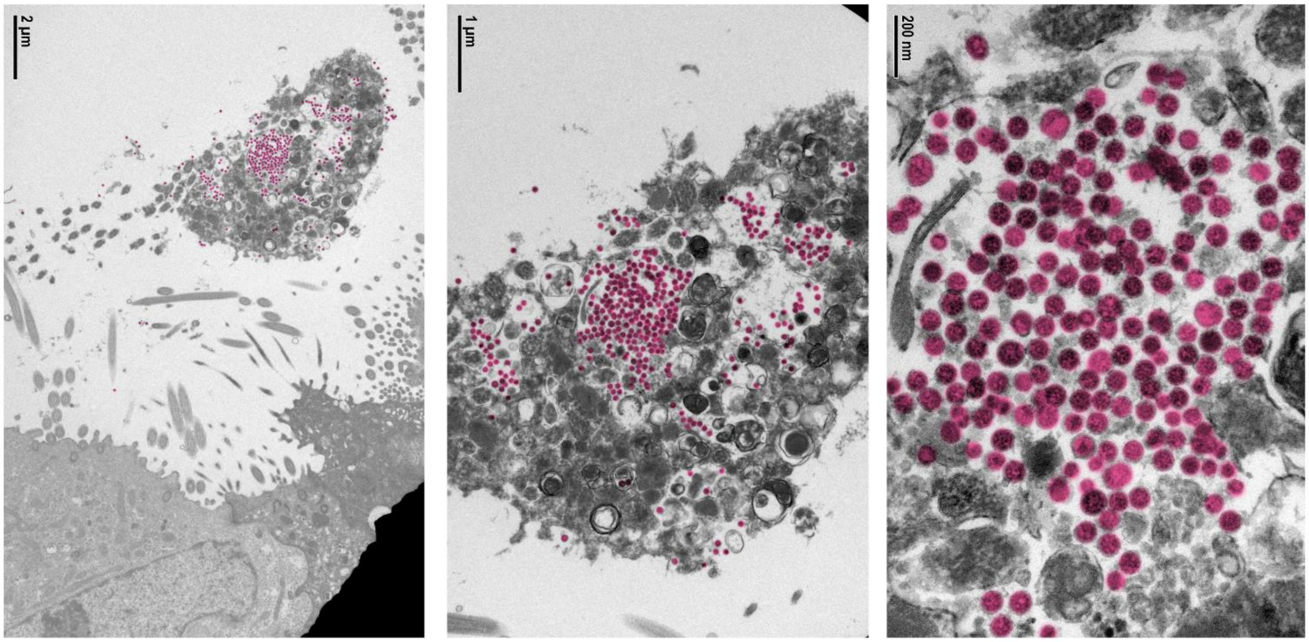

Figure S7. Colorized TEM images to illustrate viral clustering in a shed ciliated cell. Images show the late stage of anoikis from an infected ciliated cells containing virus-laden vacuoles. Viruses were colorized in pink using Adobe Photoshop to facilitate virion visualization. Scale bars are indicated on micrographs.

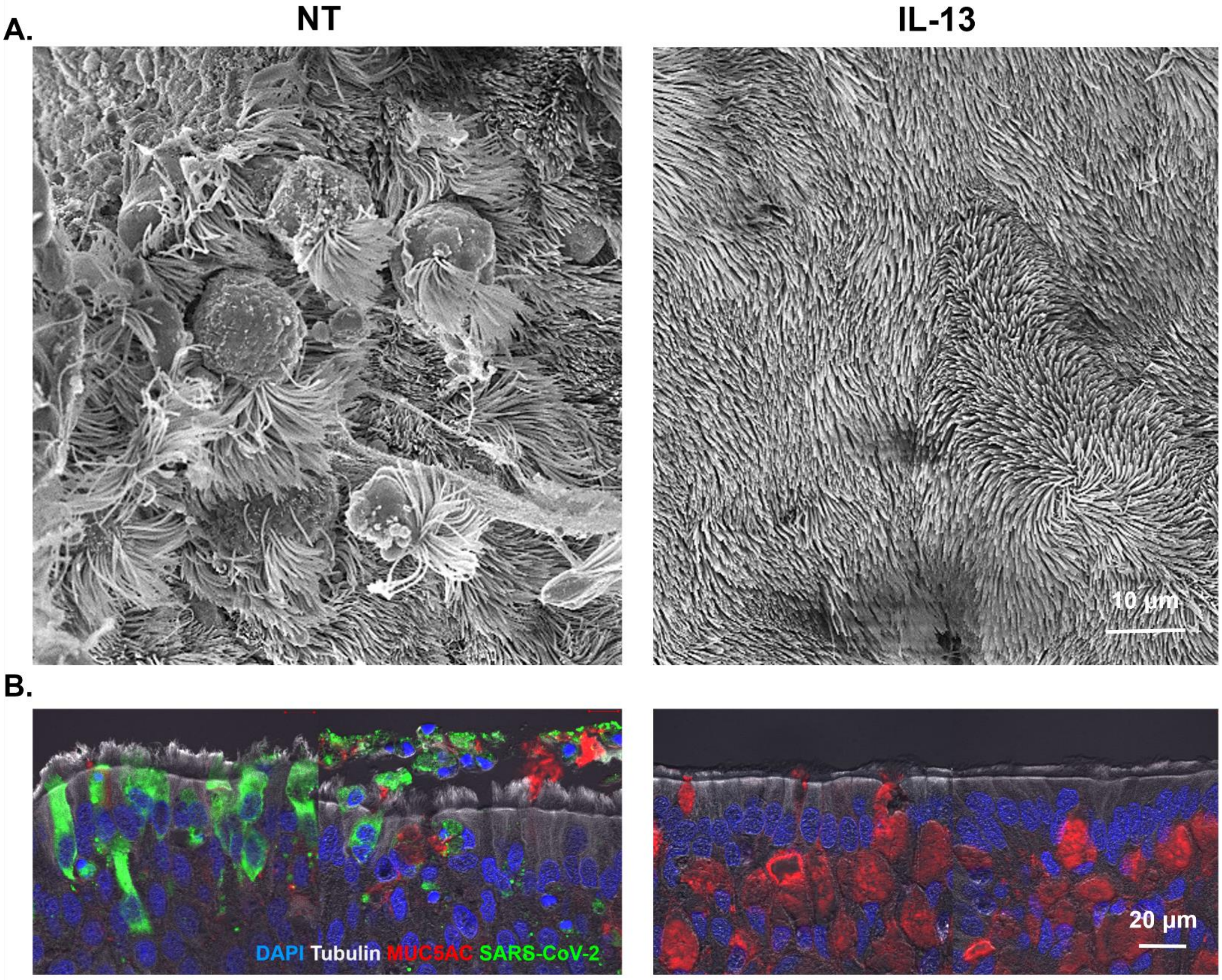

Figure S8. High magnification SEM and IHC images of NT and IL-13-treated infected HAE cells. A.) SEM showing the cell surfaces of NT (left) and IL-13-treated (right) HAE cultures infected with SARS-CoV-2 at 96 hpi. Viral shedding, cell swelling, and detachment was observed in the NT group, while epithelial damage was marginal in the IL-13-treated cells. **B.)** Merged high-magnification images of infected HAE cultures immunostained with DAPI (blue), tubulin (white), SARS-CoV-2 (green), and MUC5AC (red) comparing NT (left) and IL-13-treated cultures (right) at 96 hpi.

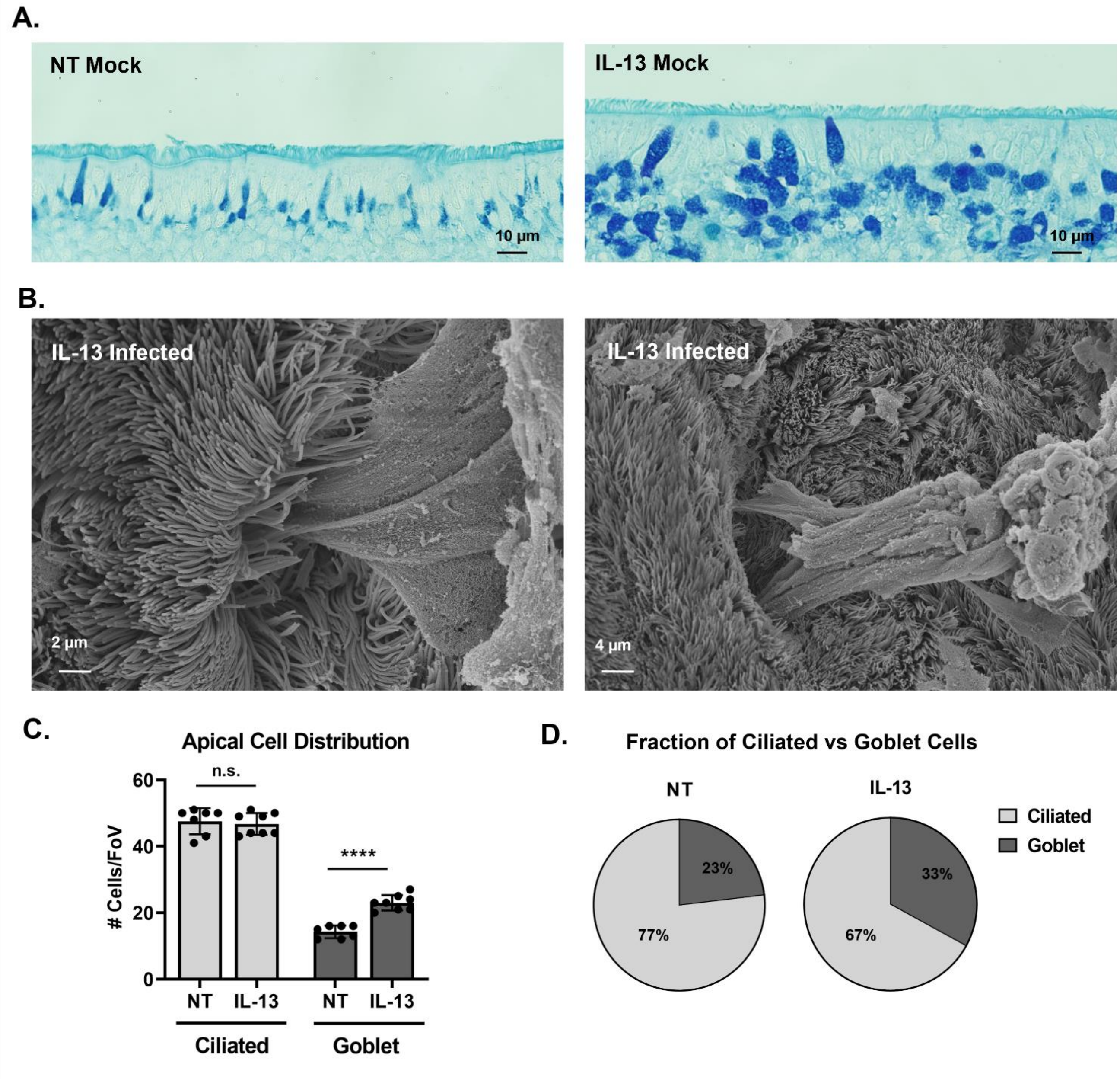

Figure S9. Apical cell type distribution in NT and IL-13-treated HAE cultures. A.) AB-PAS staining of NT and IL-13 mock cultures, showing goblet cells inserted between and underneath a layer of ciliated cells. **B.)** SEM images of SARS-CoV-2 infected IL-13-treated HAE cell cultures, depicting mucus release from hollow goblet cells surrounded by ciliated cells. **C.)** Graph indicates the number of ciliated and goblet cells per field of view (FoV) at the cell surface of NT and IL-13-treated HAE cultures as detected by AB-PAS staining. p values were acquired by multiple t-test. n.s.= non-significant, ****p<0.0001. **D.)** Pie charts display the proportion of ciliated and goblet cells in NT and IL-13-treated cell cultures.

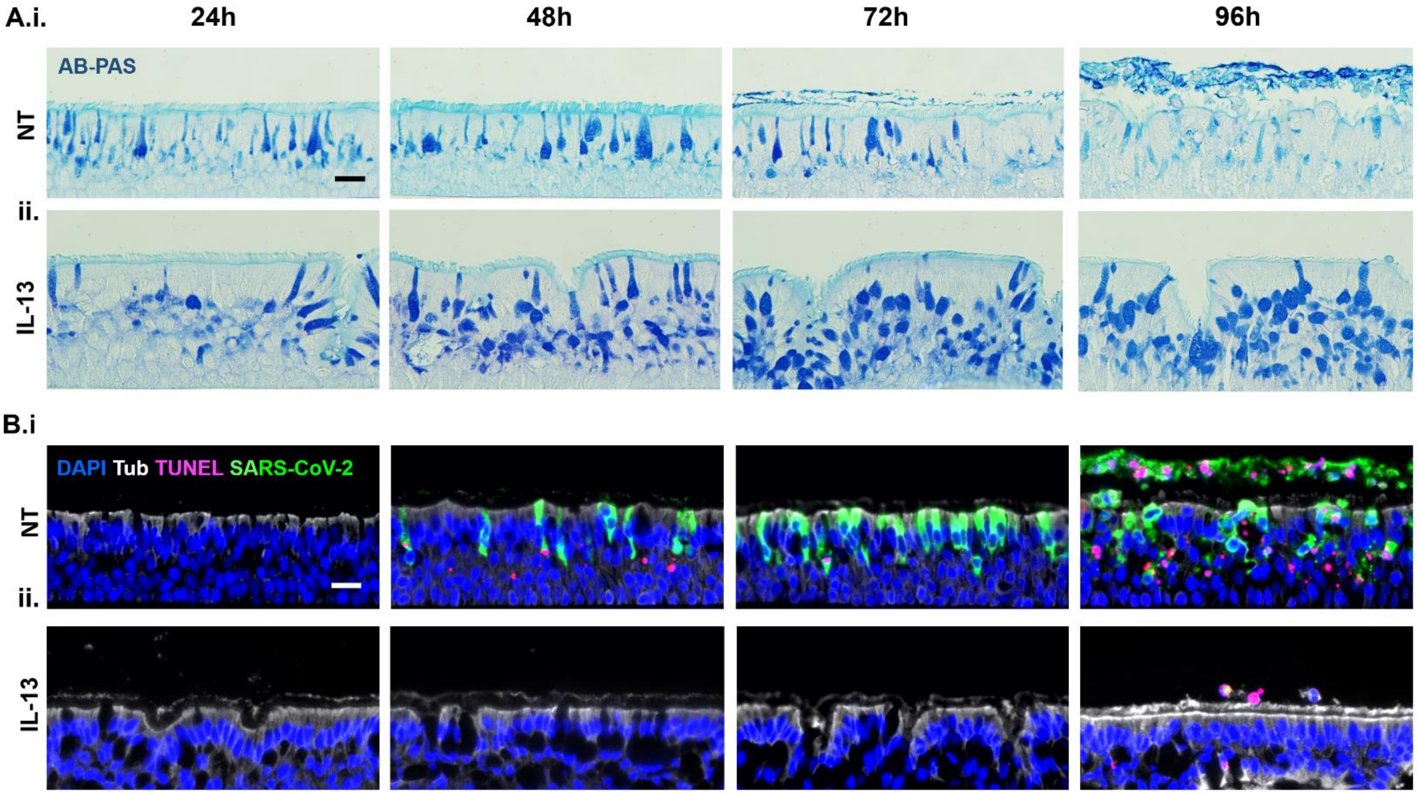

Figure S10. AB-PAS, IHC, and TUNEL staining of NT and IL-13-treated HAE cultures at 24, 48, 72, and 96 hpi. A.) Representative images of infected HAE cells at 24, 48, 72, and 96 hpi stained with AB-PAS (alcian blue-periodic acid Schiff) comparing NT **(i.)** and IL-13-treated cells **(ii.)**. **B.)** Representative images of infected HAE cell cultures at 24, 48, 72, and 96 hpi stained with DAPI (blue), tubulin (white), SARS-CoV-2 (green), and TUNEL (magenta) to examine the degree of apoptosis induced by SARS-CoV-2 infection in NT **(i.)** and IL-13 treated cultures **(ii.)**. Scale bar is 20 μm.

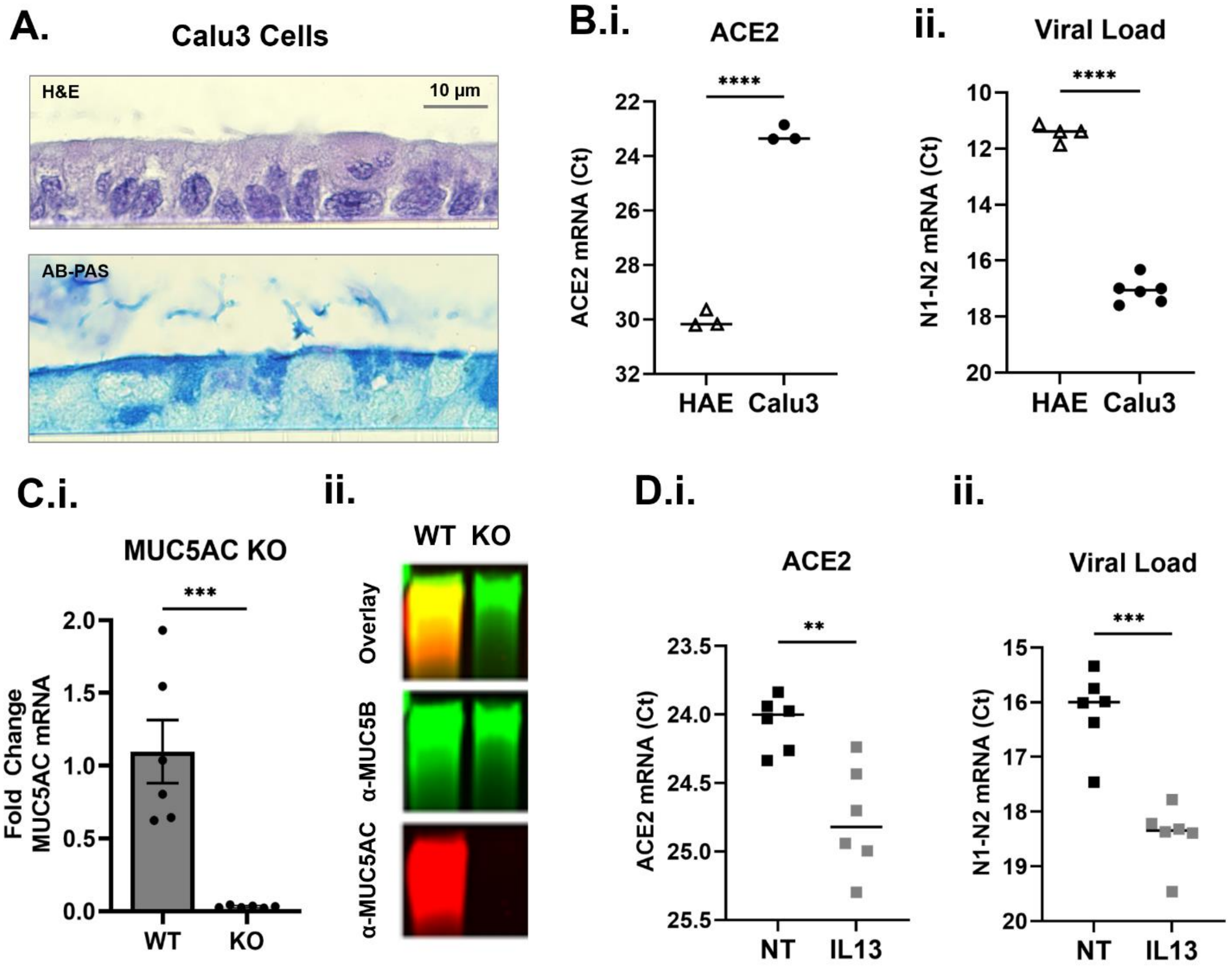

Figure S11. Effects of IL-13 on Calu3 cells knocked out for MUC5AC. A.) Calu3 cells are devoid of ciliated cells but maintain the ability to produce airway mucus. **B.)** SARS-CoV-2 infection (MOI=0.5) was performed in parallel in well-differentiated HAE and WT Calu3 cells. HAE and WT Calu3 cell mRNA were collected at 72 hpi to measure ACE2 (**i.**) and viral protein expression (**ii.**). **C.)** Validation of the MUC5AC KO Calu3 cell line via RT-PCR and Western blotting. CRISPR/CAS9 deletion approach was used on Calu3 cells to knock out MUC5AC mRNA expression (**i.**). Mucin agarose gel on cell washings from wild-type (WT) and MUC5AC KO (KO) Calu3 cells confirmed the absence of MUC5AC protein in the KO cell line by using an anti-MUC5AC (red) antibody in combination with an anti-MUC5B (green) antibody (**ii.**). **D.)** Treatment of the MUC5AC KO cell line with 1 ng/ml of IL-13 for 7 days decreased ACE2 mRNA expression (**i.**), as well as viral load as measured by N1-N2 mRNA expression (**ii.**). Data are shown as crosspoint threshold (Ct) normalized to GAPDH. n=3-6 per group. p values were acquired by student t-test. $^{**}p<0.01$, $^{***}p<0.001$, $^{****}p<0.0001$.

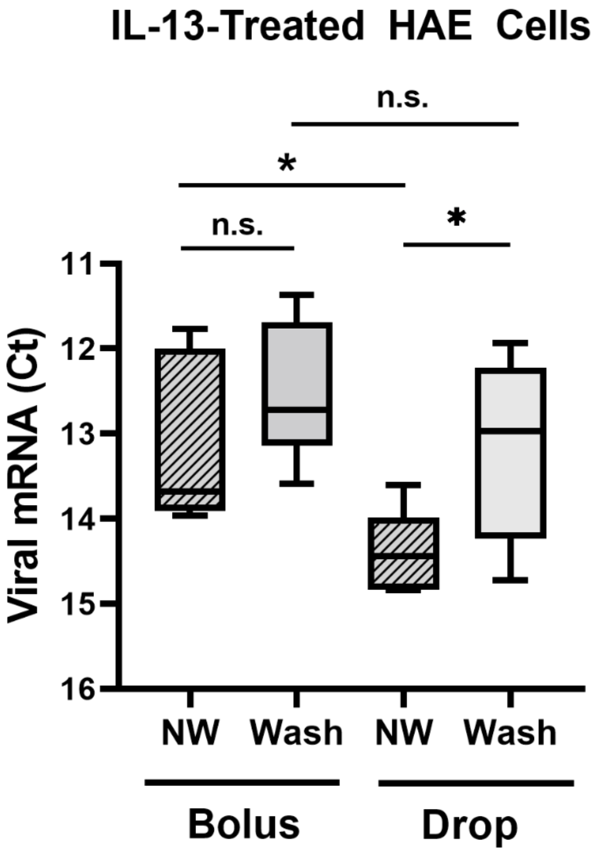

Figure S12. Effect of mucus removal on IL-13-treated cells infected with bolus and drop methods. IL13-treated HAE cells were infected with the same number of viral particles (equal inoculum) via bolus (200 µl) or drop (10 µl) methods. Bolus volumes were removed after 2h, whereas drop remained on the surface. Before infection, apical surfaces were not washed (NW) or washed (Wash) with TCEP (1mM for 15 min) and rinsed 3x with PBS to remove accumulated mucus. In the washed group, mucus was washed daily with PBS to prevent reformation of a thick mucus layer. Samples were collected for all groups at 48 hpi to measure viral mRNA expression. p values were acquired by multiple t-test. n.s.= non-significant, $^*p<0.05$.

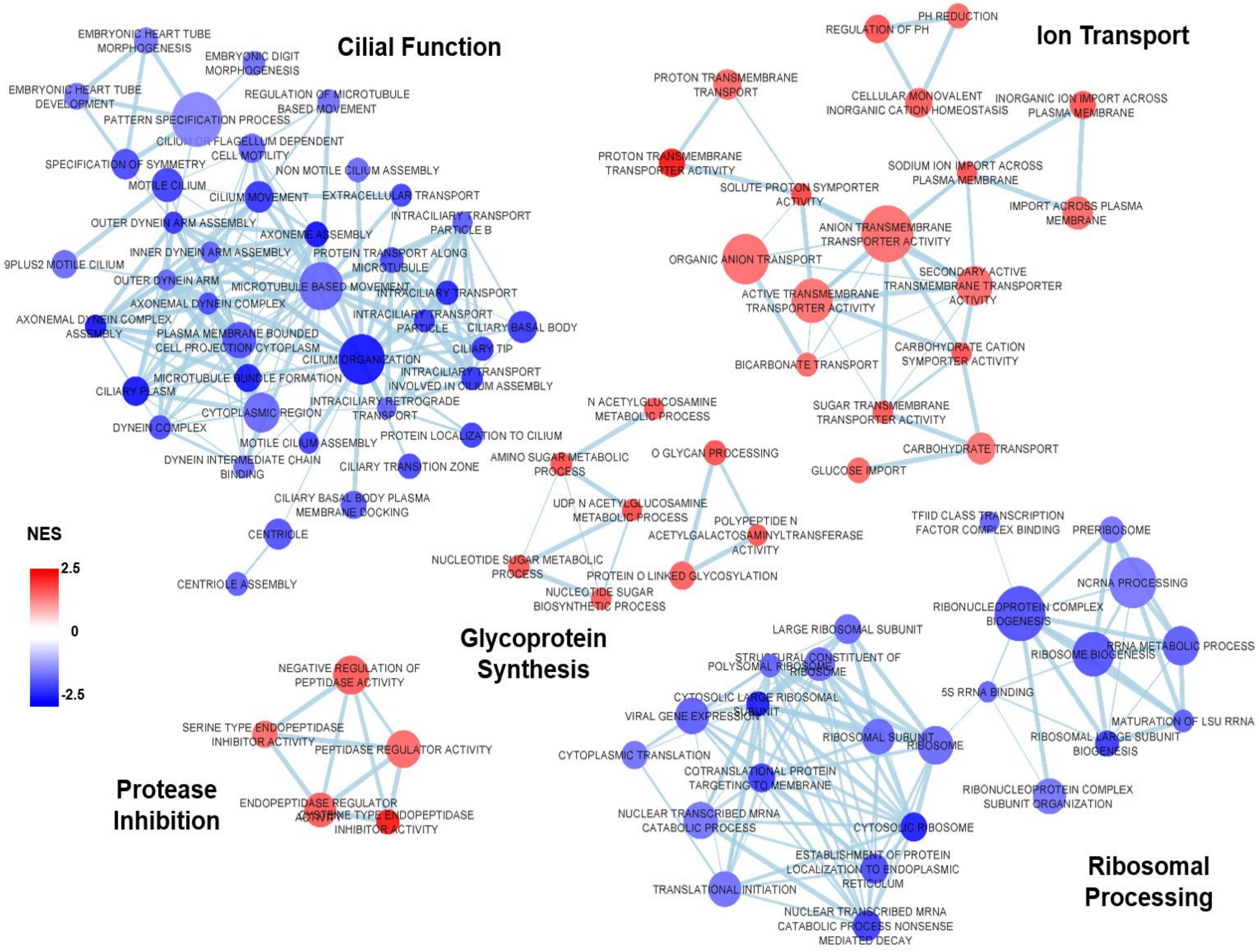

Figure S13. Detailed enrichment map of gene sets significantly altered by IL-13 treatment. Gene sets altered by IL-13 treatment of primary HAE cell cultures were downloaded from NCBI GEO datasets (GSE106812, GSE110799, and GSE37693) for meta-analysis of biological processes involved in viral proliferation. Detailed Cytoscape networks as shown in **Figure 6**, providing GO terms for affected gene sets. Raw expression data was mapped to gene symbols and transformed to log2 fold-change (log2FC) values using GEO2R for comparison across studies. Data shown as normalized enrichment score (NES) of gene sets with a false discovery rate q-value (FDR) <0.25.

A.

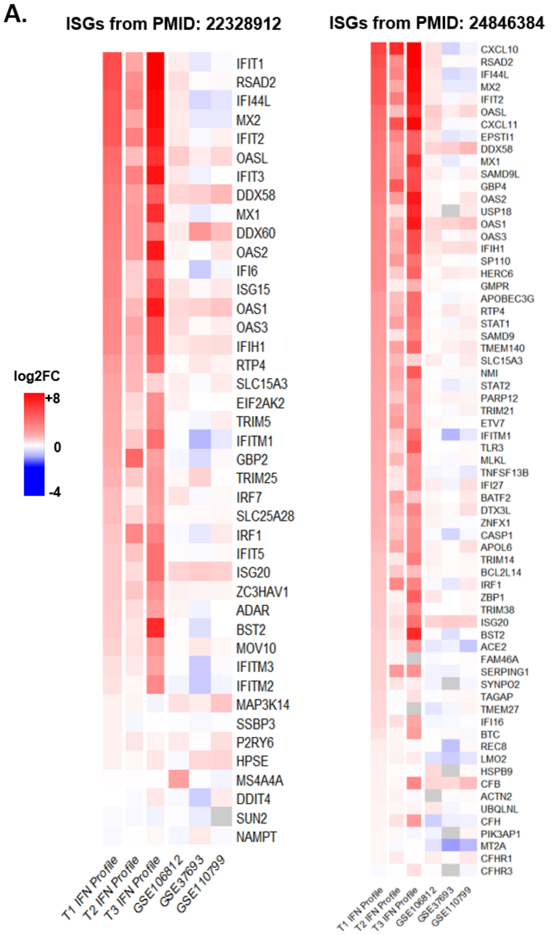

B.

NAME	NES	FDR
POSITIVE REGULATION OF INTERFERON ALPHA PRODUCTION	1.7674875	0.24186493
POSITIVE REGULATION OF INTERFERON BETA PRODUCTION	1.6759492	0.28629082
RESPONSE TO INTERFERON GAMMA	1.6051577	0.2981096
REGULATION OF RESPONSE TO INTERFERON GAMMA	1.5724739	0.3075265
INTERFERON ALPHA PRODUCTION	1.5266099	0.32159865
INTERFERON GAMMA PRODUCTION	1.3751311	0.36736557
POSITIVE REGULATION OF TYPE I INTERFERON PRODUCTION	1.2909409	0.4071943
NEGATIVE REGULATION OF INTERFERON GAMMA PRODUCTION	1.289745	0.4071971
POSITIVE REGULATION OF INTERFERON GAMMA PRODUCTION	1.2085265	0.455168
RESPONSE TO TYPE I INTERFERON	1.1933435	0.4624534
INTERFERON BETA PRODUCTION	1.1459578	0.5025909
TYPE I INTERFERON PRODUCTION	0.9438626	0.7155553
NEGATIVE REGULATION OF TYPE I INTERFERON MEDIATED SIGNALING PATHWAY	-0.62264097	0.98212415
CELLULAR RESPONSE TO INTERFERON ALPHA	-0.7410118	0.9430408
REGULATION OF TYPE I INTERFERON MEDIATED SIGNALING PATHWAY	-0.77741456	0.93419087
NEGATIVE REGULATION OF TYPE I INTERFERON PRODUCTION	-0.7863484	0.9296379
POSITIVE REGULATION OF TYPE I INTERFERON MEDIATED SIGNALING PATHWAY	-1.0500324	0.6917204
CELLULAR RESPONSE TO INTERFERON BETA	-1.071325	0.6839014
INTERFERON GAMMA MEDIATED SIGNALING PATHWAY	-1.1005616	0.6533648
NEGATIVE REGULATION OF INTERFERON BETA PRODUCTION	-1.275181	0.5018354
RESPONSE TO INTERFERON ALPHA	-1.5201118	0.31396076
RESPONSE TO INTERFERON BETA	-1.7830497	0.10851532

Figure S14. Expression data in primary HAE cells comparing interferon-stimulated genes (ISGs) with IL-13 treatment. A.) Meta-analysis of top regulated ISGs from type 1 (GSE19182), and type 2-3 (GSE153428) interferon-induced genes compared with the effects of IL-13 treatment (GSE106812, GSE110799, and GSE37693) on the same genes. All datasets were downloaded from the NCBI GEO database and published in (12, 13). Raw expression data was transformed to log2 fold-change (log2FC) values using GEO2R for comparison across studies. Genes absent from one study are represented in gray. **B.)** Effects of IL-13 treatment on interferon-stimulated GO pathways. Gene set enrichment analysis of IL-13 treatment was performed using Gene Ontology gene sets and showed equal distribution between up and downregulated GO pathways. Data shown as normalized enrichment score (NES) and false discovery rate q-value (FDR); gene sets with FDR<0.25 are highlighted in green, revealing two GO gene sets significantly altered by IL-13 in opposite direction.

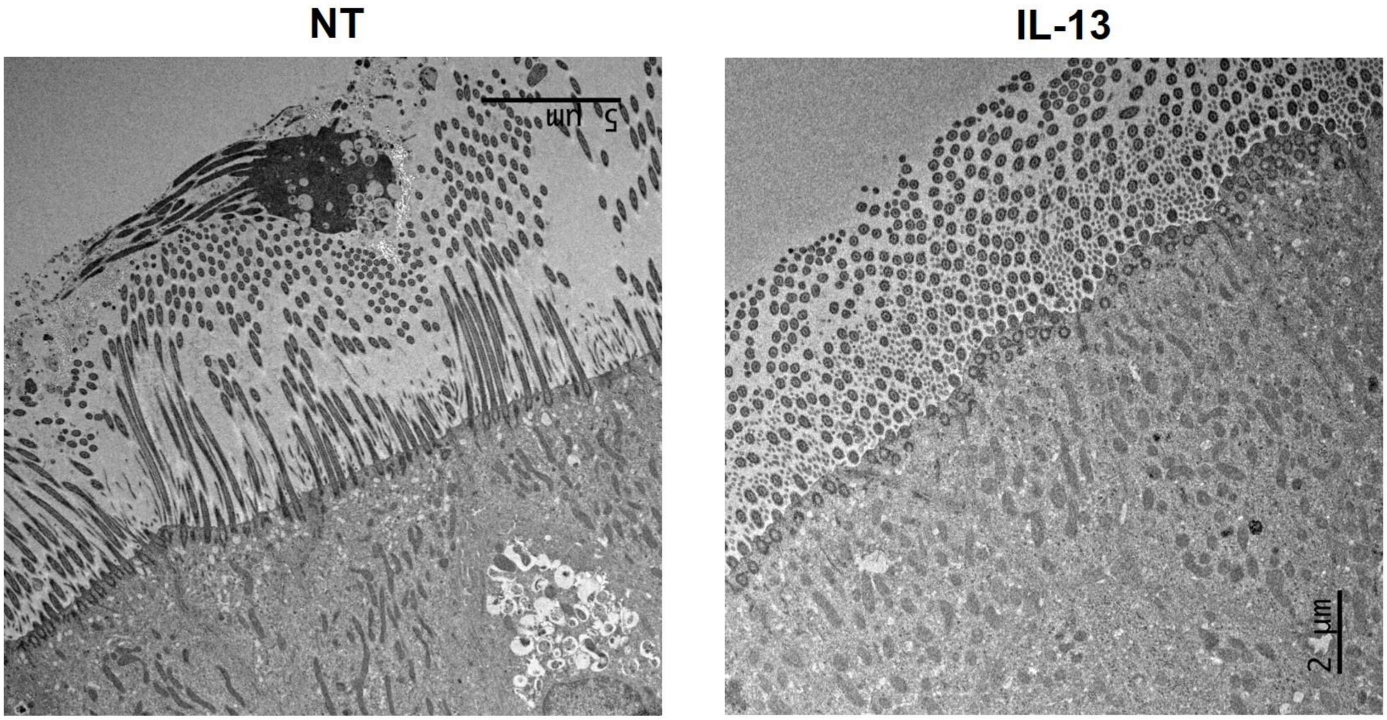

Figure S15. Low magnification TEM images of SARS-CoV-2 infected HAE cells of NT and IL-13-treated HAE cells. Representative TEM images of infected HAE cells at 96 hpi for NT (left) and IL-13-treated cells (right). In contrast with IL-13-treated cultures, cell shedding and intracellular vacuoles were hallmarks of the NT infected HAE cells. Scale bars are indicated on the micrographs.

References:

1. M. L. Fulcher, S. H. Randell, Human nasal and tracheo-bronchial respiratory epithelial cell culture. *Methods Mol Biol* **945**, 109-121 (2013).
2. G. Chen *et al.*, IL-1beta dominates the promucin secretory cytokine profile in cystic fibrosis. *J Clin Invest* **129**, 4433-4450 (2019).
3. Y. J. Hou *et al.*, SARS-CoV-2 Reverse Genetics Reveals a Variable Infection Gradient in the Respiratory Tract. *Cell* **182**, 429-446 e414 (2020).
4. X. Lu *et al.*, US CDC Real-Time Reverse Transcription PCR Panel for Detection of Severe Acute Respiratory Syndrome Coronavirus 2. *Emerg Infect Dis* **26** (2020).
5. K. A. Ramsey, Z. L. Rushton, C. Ehre, Mucin Agarose Gel Electrophoresis: Western Blotting for High-molecular-weight Glycoproteins. *J Vis Exp* 10.3791/54153 (2016).
6. C. Ehre, SARS-CoV-2 Infection of Airway Cells. *N Engl J Med* **383**, 969 (2020).
7. C. Ehre *et al.*, Overexpressing mouse model demonstrates the protective role of Muc5ac in the lungs. *Proc Natl Acad Sci U S A* **109**, 16528-16533 (2012).
8. Y. J. Hou *et al.*, SARS-CoV-2 D614G variant exhibits efficient replication ex vivo and transmission in vivo. *Science* **370**, 1464-1468 (2020).
9. C. Gene Ontology, The Gene Ontology resource: enriching a GOld mine. *Nucleic Acids Res* **49**, D325-D334 (2021).
10. M. Ashburner *et al.*, Gene ontology: tool for the unification of biology. The Gene Ontology Consortium. *Nat Genet* **25**, 25-29 (2000).
11. P. Shannon *et al.*, Cytoscape: a software environment for integrated models of biomolecular interaction networks. *Genome Res* **13**, 2498-2504 (2003).
12. V. D. Menachery *et al.*, Pathogenic influenza viruses and coronaviruses utilize similar and contrasting approaches to control interferon-stimulated gene responses. *mBio* **5**, e01174-01114 (2014).
13. J. W. Schoggins, C. M. Rice, Interferon-stimulated genes and their antiviral effector functions. *Curr Opin Virol* **1**, 519-525 (2011).
14. L. Giovannini-Chami *et al.*, Distinct epithelial gene expression phenotypes in childhood respiratory allergy. *Eur Respir J* **39**, 1197-1205 (2012).
15. K. Salka *et al.*, IFN Stimulates ACE2 Expression in Pediatric Airway Epithelial Cells. *Am J Respir Cell Mol Biol* **64**, 515-518 (2021).
16. C. B. Morrison *et al.*, Treatment of cystic fibrosis airway cells with CFTR modulators reverses aberrant mucus properties via hydration. *Eur Respir J* 10.1183/13993003.00185-2021 (2021).
